# Diverse and mobile: eccDNA‐based identification of carrot low‐copy‐number LTR retrotransposons active in callus cultures

**DOI:** 10.1111/tpj.15773

**Published:** 2022-05-10

**Authors:** Kornelia Kwolek, Patrycja Kędzierska, Magdalena Hankiewicz, Marie Mirouze, Olivier Panaud, Dariusz Grzebelus, Alicja Macko‐Podgórni

**Affiliations:** ^1^ Department of Plant Biology and Biotechnology, Faculty of Biotechnology and Horticulture University of Agriculture in Krakow 31 120 Krakow Poland; ^2^ Laboratoire Génome et Développement des Plantes, UMR 5096 CNRS/UPVD Université de Perpignan Via Domitia, 52 Avenue Paul Alduy 66 860 Perpignan Cedex France; ^3^ IRD, EMR IRD‐CNRS‐UPVD ‘MANGO’ Université de Perpignan Perpignan France

**Keywords:** *Daucus carota*, extrachromosomal circular DNA, active retrotransposons, long terminal repeats, LTR‐RTs, transposition

## Abstract

Long terminal repeat retrotransposons (LTR‐RTs) are mobilized via an RNA intermediate using a ‘copy and paste’ mechanism, and account for the majority of repetitive DNA in plant genomes. As a side effect of mobilization, the formation of LTR‐RT‐derived extrachromosomal circular DNAs (eccDNAs) occurs. Thus, high‐throughput sequencing of eccDNA can be used to identify active LTR‐RTs in plant genomes. Despite the release of a reference genome assembly, carrot LTR‐RTs have not yet been thoroughly characterized. LTR‐RTs are abundant and diverse in the carrot genome. We identified 5976 carrot LTR‐RTs, 2053 and 1660 of which were attributed to *Copia* and *Gypsy* superfamilies, respectively. They were further classified into lineages, families and subfamilies. More diverse LTR‐RT lineages, i.e. lineages comprising many low‐copy‐number subfamilies, were more frequently associated with genic regions. Certain LTR‐RT lineages have been recently active in *Daucus carota*. In particular, low‐copy‐number LTR‐RT subfamilies, e.g. those belonging to the *DcAle* lineage, have significantly contributed to carrot genome diversity as a result of continuing activity. We utilized eccDNA sequencing to identify and characterize two *DcAle* subfamilies, *Alex1* and *Alex3*, active in carrot callus. We documented 14 and 32 *de novo* insertions of *Alex1* and *Alex3*, respectively, which were positioned in non‐repetitive regions.

## Significance Statement

LTR‐RTs have significantly contributed to carrot genome evolution and diversity within the last million years and low‐copy‐number subfamilies have played a particularly important role. Extrachromosomal copies of mobilized LTR‐RTs are circularized, and hence extrachromosomal circular DNA (eccDNA) sequencing provides a means to screen for active transposable elements. In this way, few low‐copy‐number LTR‐RTs were found to be mobilized in carrot callus cultures and new copies inserted in non‐repetitive regions of the carrot genome were revealed.

## INTRODUCTION

Transposable elements (TEs) are capable of insertion into new genomic positions in a process called transposition. TEs are divided into classes, depending on the mechanism of transposition, sequence similarities and structural features (Wicker et al., 2007). Retrotransposons (class I) transpose via a ‘copy and paste’ pathway, i.e. an RNA intermediate is reverse‐transcribed and a new copy is integrated with the host genome. DNA transposons (class II) are mobilized directly via ‘cut and paste’ or ‘rolling circle’ mechanisms. TEs make up a large fraction of plant genomes, ranging from approximately 8% of the Arabidopsis genome (Quesneville, 2020) to 75% of the *Zea mays* (maize) genome (Schnable et al., 2009). Mining for TEs in newly sequenced plant genomes has revealed a substantial diversity of TEs and their high evolutionary dynamics (Borredá et al., 2019; Neumann et al., 2019; Stritt et al., 2020).

Class‐I elements are also included in the classification of the International Committee on the Taxonomy of Viruses (ICTV), through their relationship with retroviruses (Lefkowitz et al., 2018). Following the ICTV nomenclature, plant long terminal repeat retrotransposons (LTR‐RTs) are divided into Pseudoviridae (*Ty1*–*Copia* superfamily) and Metaviridae (*Ty3*–*Gypsy* superfamily). Their similarity to retroviruses is also noticeable in the process of LTR‐RT mobilization. LTR‐RTs carry a region coding for proteins essential for transposition, i.e. capsid‐related protein (GAG), protease (PR), integrase (IN), reverse transcriptase (RT) and ribonuclease H (RH). The transposition of an LTR‐RT element starts with the transcription of an active copy initiated by a promoter localized within the 5′‐LTR. Subsequently, transcripts are transported to the cytoplasm, where translation occurs. RNA templates are encapsulated into virus‐like particles (VLPs) and reverse‐transcribed. Finally, newly formed extrachromosomal LTR‐RT copies return to the nucleus and insert into new chromosomal positions (Chang et al., 2013; Schulman, 2013). Thus, each successful transposition event increases the number of LTR‐RT copies in the host genome. They are the most abundant type of TEs in plants (Benachenhou et al., 2013) and significantly contribute to the size of plant genomes (Bousios et al., 2012; Bousios & Darzentas, 2013). The two LTR‐RT superfamilies are further divided into families and subfamilies, as reflected by the phylogenies of the protein domains combined with their structural features and LTR sequences (Neumann et al., 2019).

In order to protect genome integrity, plant genomes have developed regulatory mechanisms to effectively control TEs (Paszkowski, 2015). The activation of LTR‐RTs may be triggered by a range of abiotic or biotic stresses (Dubin et al., 2018; Grandbastien, 1998; Lanciano & Mirouze, 2018; Wessler, 1996). Some LTR‐RT promoters contain sequences recognized by transcription factors involved in stress responses, e.g. the activation of heat defense pathways induces the mobilization of the *ONSEN* retrotransposon in *Arabidopsis thaliana* (Cavrak et al., 2014). Other reports documented LTR‐RT mobilization in response to abiotic stresses (Butelli et al., 2012; Ito et al., 2013; Pietzenuk et al., 2016; Sahin et al., 2020), pathogen elicitors (Anca et al., 2014; Pouteau et al., 1994) and plant hormones (He et al., 2012; Nie et al., 2019). LTR‐RTs can be mobilized *in vitro*, possibly owing to relaxation of the epigenetic control of the host. Mobilization of *Tnt1* in *Nicotiana tabacum* (tobacco) protoplasts was the first reported example of LTR‐RT activation induced by cell cultures (Grandbastien et al., 1989; Pouteau et al., 1991). Subsequently, *Tto* and *Tos* elements were demonstrated to be mobile in tissue cultures of tobacco (Hirochika, 1993) and *Oryza sativa* (rice) (Hirochika et al., 1996a), respectively. More recently, Bayram et al. (2012) identified new insertions of *Nikita* LTR‐RT in the *Hordeum vulgare* (barley) callus. As TEs are essentially selfish or parasitic (Orgel & Crick, 1980), the effects of new insertions are expected to range from deleterious to neutral to the host (Cosby et al., 2019; Kremer et al., 2020). However, a growing body of evidence indicates that some insertions could possibly provide long‐term adaptive effects at the population level (Galindo‐González et al., 2017; Lanciano & Mirouze, 2018).

In plants, extrachromosomal circular DNA (eccDNA) can be formed from tandem repeats (Cohen et al., 2008; Navrátilová et al., 2008), from LTR‐RTs (Hirochika & Otsuki, 1995) and from some class‐II transposons (Sundaresan & Freeling, 1987). eccDNAs originating from LTR‐RTs have been extensively analyzed in yeast, showing that they mostly contained full‐length elements. Møller et al. (2015) pointed to the possibility of the relocation of *Ty1* retrotransposons in yeast directly through DNA circularization, without an RNA intermediate. However, Lanciano et al. (2017) proposed that the formation of LTR‐RT‐derived eccDNAs should instead be viewed as a mechanism limiting the number of new insertions of active retrotransposons. Based on that assumption, they proposed a strategy aiming at the identification of active LTR‐RTs in plant genomes by means of eccDNA sequencing.

Carrot (*Daucus carota* ssp. *sativus*, 2*n* = 2*x* = 18) is a diploid species belonging to the Apiaceae family. The size of the carrot genome is 473 Mb and a high‐quality genome reference assembly of a doubled haploid line (DH1) derived from an orange Nantes‐type cultivar is available (Iorizzo et al., 2016). TEs comprise almost 200 Mb of the carrot genome, of which approximately 70% is attributed to class‐I elements. However, carrot LTR‐RTs have not been investigated in more detail. Here, we describe a landscape of LTR‐RTs in the carrot genome, provide their comprehensive annotation and description, and present evidence for the mobilization of certain low‐copy‐number *Copia* subfamilies in callus cultures.

## RESULTS

### LTR‐RT landscape in the carrot genome

We identified 5976 LTR‐RT copies flanked by LTRs and target‐site duplications (TSDs), as reported by ltrharvest (Table S1). Approximately 80% (4738) copies were localized on assembled chromosomes of the DH1 carrot reference genome. Of those, 1849 copies were not classified and were removed for downstream analysis. Hence, we focused on 3713 copies unambiguously classified into the *Copia* or *Gypsy* superfamilies and localized on the assembled chromosomes. They represented lineages usually recognized in plant genomes (Table 1).

**Table 1:** LTR‐RT lineages identified on the assembled chromosomes of the DH1 carrot reference genome

LTR retrotransposon lineage	Masked (bp)	Masked (%)	LTRharvest number of intact copies	LTRharvest copies (bp)	Copies/masked ratio	Age (Myr)
*Copia/DcAle*	4 788 879	1.14%	241	1 505 278	31.43%	1.14
Copia*/DcAlesia*	342 801	0.08%	8	55 937	16.32%	1.44
*Copia/DcAngela*	4 840 815	1.15%	115	1 878 695	38.81%	1.38
*Copia/DcBianca*	1 733 098	0.41%	91	610 250	35.21%	0.82
*Copia/DcIkeros*	208 348	0.05%	7	62 630	30.06%	1.26
*Copia/DcIvana*	5 814 811	1.38%	154	1 350 124	23.22%	1.20
*Copia/DcSIRE*	37 892 761	9.00%	897	13 239 278	34.94%	1.70
*Copia/DcTAR*	1 637 944	0.39%	104	737 471	45.02%	1.21
*Copia/DcTork*	815 871	0.19%	41	289 322	35.46%	0.88
*Gypsy/DcAthila*	10 401 694	2.47%	309	3 820 029	36.73%	1.61
*Gypsy/DcCRM*	389 629	0.09%	16	111 581	28.64%	0.84
*Gypsy/DcGaladriel*	45 340	0.01%	2	14 799	32.64%	1.15
*Gypsy/DcReina*	2 143 499	0.51%	146	912 136	42.55%	0.64
*Gypsy/DcRetand*	19 321 421	4.59%	493	7 187 972	37.20%	1.24
*Gypsy/DcTekay*	9 151 566	2.17%	265	3 454 491	37.75%	1.57
unclassified	101 905 289	24.18%	1849	17 955 034	17.62%	2.16
total	201 433 766	47.81%	4738	53 185 027	–	–

**Figure 1:**
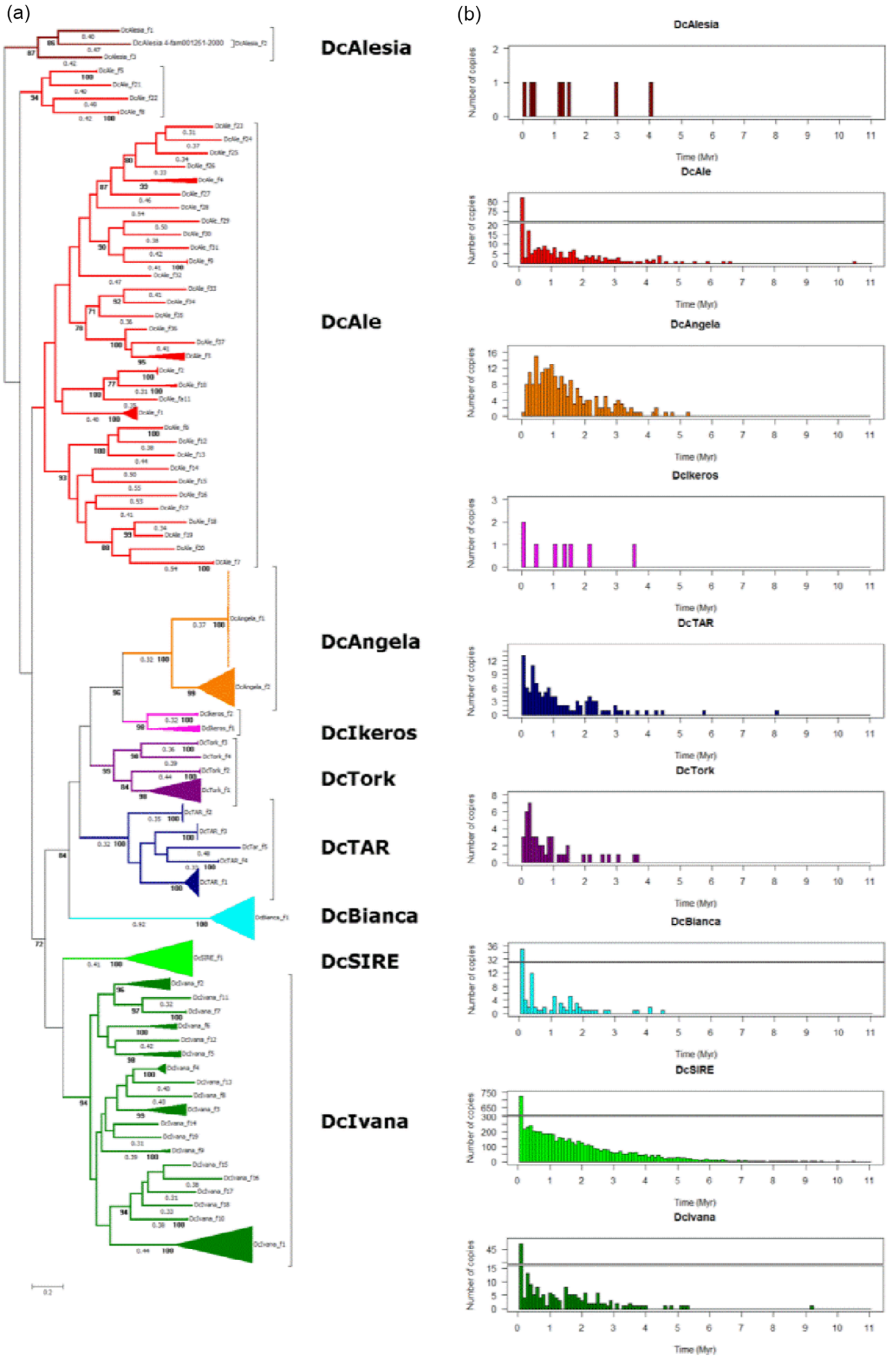
(a) Maximum‐likelihood tree of *Copia* elements, based on retrotransposon (RT) domains of 441 copies. (b) Distribution of insertion times (in 0.1‐Myr bins) of all elements belonging to the *Copia* lineages. Branch length (numbers below branches) represent the number of substitutions per site. Branch support was estimated with 1000 bootstrap replicates (numbers below branches, in bold). [Colour figure can be viewed at wileyonlinelibrary.com]

Overall, LTR‐RT elements comprised almost 48% of the carrot reference genome. The group of ‘unclassified’ LTR‐RTs (i.e. those not attributed unambiguously to either *Gypsy* or *Copia* superfamilies) occupied almost 24% of the assembled genome, including 1849 copies flanked by LTRs. However, they were on average much older than any of the *Copia* or *Gypsy* lineages (Table 1). The *Copia* superfamily accounted for 13.79% of the assembled genome, whereas 9.84% was attributed to the *Gypsy* superfamily. *Copia* LTR‐RTs were slightly more numerous than *Gypsy* LTR‐RTs in terms of the number of intact elements, comprising 2053 and 1660 copies, occupying 4.17% and 3.28% of the genome, respectively (Table S1). The *Copia* superfamily was also more diverse than the Gypsy superfamily (Figures 1, S1 and S2). Subsequently, we followed the classification of carrot LTR‐RTs into lineages, using a strategy proposed generally for plant retrotransposons by Neumann et al. (2019).

The LTR‐RT families were defined based on the reverse transcriptase (*rt*) domain phylogeny and labeled consecutively from ‘f1’ to ‘fn’, whereas truncated elements not grouping with any complete copy were combined into one artificial family per lineage and labeled ‘f0’. The most diverse *Copia* lineages, *DcAle* and *DcIvana*, were divided into 38 and 20 families, respectively. Four lineages, *DcAngela*, *DcAlesia*, *DcTork* and *DcTar*, grouped three, four, five and six families, respectively, whereas each of the remaining three lineages, *DcBianca*, *DcIkeros* and *DcSIRE*, comprised a single family. In contrast, the most diverse Gypsy lineage *DcAthila* comprised five families, *DcRetand*, *DcReina* and *DcCRM* included a single family each, whereas no representative copy meeting the criteria for phylogenetic analysis was revealed within *DcTekay*, hence all copies of that lineage were attributed to f0. However, although the estimated mean age of *DcTekay* elements (1.57 Myr) was relatively high compared with other lineages (Table S2), the distribution of insertion times of the lineage showed that certain copies might have been active recently, as 20 copies carried identical LTRs (Data S1).

We further grouped all LTR‐RT copies into subfamilies, producing a total of 1140 subfamilies, 588 and 552 of which were attributed to *Copia* and *Gypsy*, respectively (Figure S3, Figure_S3.html)). Nearly half (46.2%) of the LTR‐RT copies mapping to the assembled carrot chromosomes were grouped into low‐copy‐number subfamilies, comprising from one to 10 copies (Figure S3; Table S2). The most numerous LTR‐RT lineages were usually characterized by the low incidence of low‐copy‐number subfamilies and high solo LTR/intact LTR‐RT ratio (Table S2). This was reflected by a strong correlation between the number of copies per lineage with the number of solo LTRs produced by that lineage (*R* ^2^ = 0.93, *P* = 1.44e‐5). Also, the number of solo LTRs correlated with the mean age of the lineage (*R* ^2^ = 0.67, *P* = 1.69e‐2) (Figure S4).

The most numerous lineages (*DcSIRE*, *DcRetand* and *DcAthila*) contained relatively few single‐copy subfamilies (9%, 18% and 19%, respectively) and showed high solo LTR/intact LTR‐RT ratios (6.36, 2.14 and 4.57, respectively). Interestingly, the other four lineages with more than 140 copies, *DcTekay* (*Gypsy*), *DcAle* (*Copia*), *DcIvana* (*Copia*) and *DcReina* (*Gypsy*), showed higher frequencies of single‐copy subfamilies, ranging from 48% to 65%. In particular, *DcAle* was the most diverse lineage, comprising 174 subfamilies with no more than eight copies per subfamily. Moreover, all but one of those four lineages were characterized by low solo LTR/intact LTR‐RT ratio (below 0.61), whereas for *DcTekay* the ratio was much higher (3.82). The abundance of solo LTRs in *DcTekay* might be explained by the estimated age and fact that these subfamilies carried longer LTRs and had a larger proportion of LTR length to total length of TE (Data S2), as shown for other LTR‐RTs in plant genomes (Vitte et al., 2007).

In general, detectable carrot LTR‐RTs spanned a period of 4 Myr, with a mean age of the lineage varying from 0.6 to 1.7 Myr (Figure S5). The most recent insertions, less than 1 Myr old, occupied 3.36% of the carrot genome (1.77% of *Copia* and 1.58% of *Gypsy*) and elements that were active 1–2 Mya accounted for 2.06% of the genome (1.21% of *Copia* and 0.85% of *Gypsy*), whereas the remaining copies constituted 2.03% of the genome (1.19% of Copia and 0.84% of Gypsy). Nevertheless, even in the older lineages, such as *DcSIRE* (*Copia*) and *DcAthila* (*Gypsy*), copies inserted more recently (<0.1 Mya) were also observed. Also, within all but one lineage, copies attributed to f0, i.e. those not meeting the criteria required for grouping, were relatively older than other families. The only exception was f0 of *DcRetand*, apparently younger on average than the f1 family, possibly pointing to a recent proliferation of non‐autonomous copies. The youngest copies were found in *DcReina*, followed by *DcBianca*, *DcCRM*, *DcTork* and *DcAle* (Figures 1, S1 and S2).

### Genomic localization

In general, intact LTR‐RTs, as well as solo LTRs, clustered within pericentromeric regions. However, other chromosomal regions rich in intact LTR‐RTs and solo LTRs were also observed (Figure S6). No significant differences in the distribution of *Copia* and *Gypsy* copies with respect to the nearest gene were observed (1‐kb bins spanning 1–10 kb from the nearest gene, χ^2^ goodness‐of‐fit test, *P* = 0.069; two‐sample Kolmogorov–Smirnov test, *P* = 0.707).

Approximately 83% of LTR‐RT copies were localized in intergenic regions (i.e. more than 1 kb away from the closest gene). The general trend for positioning intact copies and solo LTRs from the same family was similar. Lineages comprising more diverse families with many low‐copy‐number subfamilies, in particular *DcAle* (*Copia*) and *DcReina* (*Gypsy*), were more frequently associated with genic regions, whereas the opposite relationship was characteristic for the most abundant and less diverse families *DcSIRE* (*Copia*) and *DcRetand* (*Gypsy*) (Figure S7). To verify this in more detail, we looked at genomic positions of elements classified into subfamilies containing from one to 10 copies. Indeed, a strong relationship between the copy number per subfamily and localization in the genic region was revealed (Figure S3). However, the distribution of elements belonging to low‐copy‐number subfamilies did not differ markedly from the general distribution observed for the lineages (Pearson’s χ^2^ test, *P* = 9e‐4; Figure S7c), indicating that the tendency for a closer association with genes was more lineage dependent than copy‐number dependent, and that intact LTR‐RT copies from lineages containing low‐copy‐number subfamilies were more frequently associated with genic regions.

### Active LTR retrotransposons in carrot callus cultures

#### Identification of LTR‐RT‐derived eccDNAs

Illumina sequencing libraries were produced for four carrot eccDNA samples derived from two pairs of callus sublines, derived from different donors (DH1 line and ‘Koral’ cv.). Each pair of sublines differed in terms of callus morphology: the ‘Koral’‐derived sublines, K10w and K10p, produced white and purple callus, respectively, whereas the DH1‐derived sublines, DH1py and DH1do, produced pale‐yellow and dark‐orange callus, respectively (Figure S8; Table S3). The overall alignment rate to the DH1 carrot reference genome was around 70%. Reads mapping to the database of carrot LTR‐RTs varied from 9.82% to 23.20% of the filtered paired reads for DH1py and K10p, respectively. In order not to downplay the information contained in approximately 30% of the eccDNA reads that did not map to the reference assembly spanning 421.5 Mb of the 473 Mb genome (90%), *de novo* assembly of eccDNA was performed. The assemblies, depending on the callus subline, included 554–856 contigs with N50 ranging from 1418 to 2004 bp, for DH1py and K10p, respectively (Table S4). Of those, 96 K10p contigs and 137 K10w contigs showed similarity to carrot LTR‐RTs.

We performed comparative analysis using repeatexplorer to identify eccDNAs enriched in all eccDNA libraries, as well as those enriched in individual samples. The clustering summary showed that 42% of reads represented sequences that were repetitive and enriched in eccDNA, whereas the remaining 58% reads were singlets, likely representing background genomic sequences. The largest number of clustered reads was attributed to plastid and mitochondrial DNA (22.56%), followed by the *DcAle* lineage (4.25%). As carrot *DcAle* elements span merely 0.3% of the carrot genome, the result reflects an overrepresentation of the lineage in eccDNA libraries (Table S5). We identified 41 repeatexplorer clusters annotated as LTR‐RTs, 17 of which comprised more than 500 reads, with more than 50% reads attributed to carrot LTR‐RTs (Table S6). Graphs representing individual clusters were manually inspected and clusters were merged and attributed to 10 LTR‐RT subfamilies, belonging to *DcAle*, *DcIvana*, *DcBianca*, *DcTAR* and *DcTork* lineages of the *Copia* superfamily (Figure S9; Tables S6 and S7). All those subfamilies contained from one to eight young copies (Table S8). In case of all but three subfamilies (two *DcTAR* and one *DcTork*), all conserved domains characteristic for *Copia* were detected (Table S8). It suggests that LTR‐RTs identified in eccDNA reads represent complete and likely active elements. All identified subfamilies were characterized by different proportions of eccDNA reads in pairs of callus sublines derived from the same donor (Tables S6 and S7).

#### Search for novel insertion sites

To identify LTR‐RT insertion sites, we resequenced genomic DNA of three callus sublines, K10w, K10p, and DH1do (Figure S8), using Illumina PE‐mode. The sequencing libraries comprised reads from 59 607 033 to 91 524 906, with the mapping rate to the carrot DH1 reference genome ranging from 98.35% to 99.65%, and with estimated sequencing depths of 19×, 28× and 29× for K10p, DH1do and K10w, respectively (Table S9). The DH1do callus subline was developed from a DH1 plant for which the reference genome had been assembled, whereas both K10 callus lines were developed from the same donor plant from the ‘Koral’ cv. Thus, we assumed that any LTR‐RT insertion polymorphism between the DH1 reference genome and the resequenced genome of the DH1do callus subline, or between the K10w and K10p callus sublines, reflected *de novo* transposition events that occurred over the course of callus culture.

For all LTR‐RT subfamilies forming eccDNAs in the callus sublines, insertion sites in the resequenced genomes were identified using trackposon, originally developed for the time‐efficient identification of signatures of LTR‐RT insertions in rice (Carpentier et al., 2019). We observed a striking difference in the rate of *de novo* insertions between the two pairs of callus sublines. Whereas in the white and the purple callus sublines, K10w and K10p, a number of *de novo* insertions were identified, as described in detail below, no clear indication for any novel insertions was observed in the DH1‐derived DH1do callus subline, despite the fact that LTR‐RT‐derived eccDNAs were observed in all samples.

For some subfamilies identified in eccDNA, i.e. DcAle_f0/s0191, DcBianca_f1/s1628, DcTAR_f0/s1350 and DcTAR_f0/s2199, we have not detected any *de novo* integration sites, suggesting that they might have been effectively repressed before reinsertion. For other subfamilies, between two and 32 *de novo* insertions have been identified (Tables S11–S17). However, *de novo* insertions for subfamilies *DcAle_f1/s0318* (*Alex2*), *DcIvana_f6/s0395* (*Ivan1*), *DcTork_f0/s1917* and *DcTork_f1/s2099* could not have been precisely characterized, as they were localized in repetitive regions. Notably, reference copies of those subfamilies were also present in repetitive regions. Thus, we focused on the *DcAle_f2.s0082* (*Alex1*) and *DcAle_f6.s1092* (*Alex3*) subfamilies, representing the *DcAle* lineage, and localized mostly in non‐repetitive regions. We aligned *Alex1* and *Alex3* copies present in the DH1 reference genome with contigs reconstructed from K10w and K10p eccDNA libraries to verify the completeness of LTR‐RTs and the presence of the TSDs, PPB and PPT. *Alex1* and *Alex3* copies were reconstructed from eccDNA reads, and all but one *Alex1* reference copy were complete (Datas S3 and S4).

Non‐reference insertion sites of *Alex1* and *Alex3* were found in the K10w and K10p callus sublines. Most of them were not shared between K10w and K10p (Figures 2 and S10–S17), indicating independent transposition events in the course of the callus culture.

**Figure 2:**
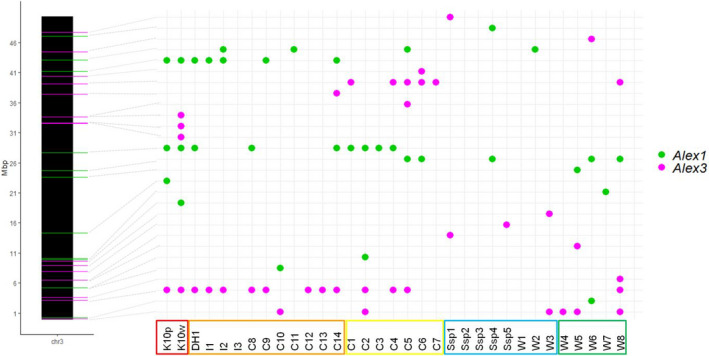
Localization of *Alex1 and Alex3* copies on carrot chromosome 3 and their distribution in cultivated and wild carrots. Groups representing K10 callus, western cultivated carrots, eastern cultivated carrots, European wild *Daucus carota* and Asian wild *D. carota* are framed in red, orange, yellow, blue and green, respectively. [Colour figure can be viewed at wileyonlinelibrary.com]

Eight copies were attributed to the subfamily *Alex1* in the carrot DH1 reference genome (Table S8). Six of those were identified upon whole‐genome resequencing of the K10w and K10p callus sublines, whereas all eight were found, as expected, in the genomes of DH1 and DH1do. In total, we identified 17 non‐reference *Alex1* copies in the K10 callus, three of which were shared by both ‘Koral’‐derived sublines, likely representing insertions present in the donor plant or *de novo* insertion that took place in callus cultures before the separation of the K10w and K10p sublines. Thus, a total of nine insertions of *Alex1* copies pre‐dated the separation of K10p and K10w, whereas eight and six insertions were unique for K10p and K10w, respectively (Figures 2 and S10–S17; Table S12). Notably, all 23 *Alex1* copies, including the reference copies, were localized in genic regions.

*Alex3* was represented by a single copy in the reference genome that was also present in both ‘Koral’‐derived callus sublines. We have not found any *Alex3* insertion shared between K10w and K10p other than the reference insertion, whereas we identified 32 *de novo* insertions (19 and 13 in K10w and K10p, respectively), most of them positioned near genes (Table S14). This points to the substantial tissue culture‐induced activity of *Alex3* in the ‘Koral’ genetic background.

### Validation of *de novo* insertion sites

#### Validation of the pipeline used to identify *de novo* insertions

As we modified the trackposon pipeline for a more precise determination of LTR‐RT insertion sites using soft‐clipped reads, we first validated its performance using the carrot DH1 reference genome before and after the masking of eight and one insertion sites of *Alex1* and *Alex3*, respectively, present in the reference genome. We reanalyzed reads obtained from the resequencing of K10w, K10p and DH1do callus sublines and reads available for the DH1 reference genome using the unmasked and masked genome. Masked reference insertions were expected to be identified as *de novo* insertions, supported by at least one soft‐clipped read. We assumed that false positives would be represented by soft‐clipped reads for the reference insertion site in the non‐masked genome, whereas false negatives would be represented by the lack of soft‐clipped reads for any reference insertion in the masked genome. In total, 36 calls (nine insertion sites and four samples) were used to estimate the rates of false positives and false negatives. We identified two positive calls of soft‐clipped reads for the non‐masked genome and one negative call of soft‐clipped reads in the masked genome that translates to 5.5% false‐positive and 2.8% false‐negative calls (Table S10). Thus, we concluded that the modified trackposon pipeline reliably identified non‐reference LTR‐RT insertion sites.

#### *In silico* identification of *Alex1* and *Alex3* insertions in 31 resequenced carrot genomes

To verify the uniqueness of the candidate *de novo* insertions described above and the general abundance of *Alex1* and *Alex3* subfamilies, we analyzed their distribution in 31 previously resequenced genomes of cultivated and wild carrots (Iorizzo et al., 2016; Tables 2 and S18), and in the ‘Koral’‐derived callus sublines. In total, we identified 78 and 99 positions harboring *Alex1* and *Alex3* copies, all of them being polymorphic in the investigated pool of genomes (Data S5; Figures 2 and S10–S17). Importantly, the *de novo* insertions revealed in K10w and K10p were not found in any of the resequenced genomes, indicating that they are likely to represent genetic novelty resulting from mobilization events that occurred in callus cultures.

**Table 2:** Number of *Alex1* and *Alex3* insertions in the DH1 reference genome, two resequenced carrot ‘Koral’‐derived callus sublines (K10w and K10p) and 30 genomes of *Daucus carota*; detailed characteristics of the resequenced genomes are provided in Table S18

ID	*Alex1* insertions	*Alex1* unique insertions	*Alex3* insertions	*Alex3* unique insertions
K10p	17	8	14	13
K10w	15	6	20	19
DH1	8	1	1	0
I1	5	0	4	0
I2	7	0	2	0
I3	8	1	2	0
C8	3	1	2	1
C9	7	3	3	0
C10	8	2	4	0
C11	6	0	2	0
C12	5	0	4	0
C13	5	0	4	0
C14	8	0	4	1
C1	5	0	3	1
C2	5	1	6	2
C3	7	0	7	4
C4	6	0	4	1
C5	8	0	6	2
C6	4	0	3	1
C7	5	1	5	2
Ssp1	6	1	6	3
Ssp2	3	1	6	2
Ssp3	2	0	5	2
Ssp4	6	2	1	0
Ssp5	6	4	7	5
W1	5	1	9	7
W2	3	0	5	3
W3	4	2	4	2
W4	6	1	3	1
W5	8	2	4	2
W6	16	11	7	6
W7	3	2	0	0
W8	8	2	8	3

The number of A*lex1 and Alex3* copies ranged from two to 17 and from zero to 20 per individual genome, with an average of seven and five copies per genome, respectively (Table 2). Both the total number of copies and the total number of unique insertion sites per plant of *Alex1* and *Alex3* were the highest in the ‘Koral’‐derived callus sublines. The only exception was a wild accession (W6), possessing a high number of *Alex1* copies (at 16 insertion sites, 11 of which were unique), possibly resulting from a recent *in vivo* mobilization of *Alex1*. The most common insertion site of *Alex1* was shared by 28 of the 33 accessions, including both ‘Koral’‐derived callus sublines (Data S5; Figures 2 and S10–S17). That insertion was positioned near an annotated gene, whereas four other *Alex1* copies and one *Alex3* copy, present in more than half of the cultivated carrots, were localized in introns or near genes. The remaining *Alex1* and *Alex3* insertion sites were less common. Although those present in the wild *D. carota* were usually found in a single accession, those found in the cultivated carrots were typically shared by more than one accession (Data S5; Figures 2 and S10–S17; Table 2).

#### PCR‐based validation of *de novo* insertion sites

**Figure 3:**
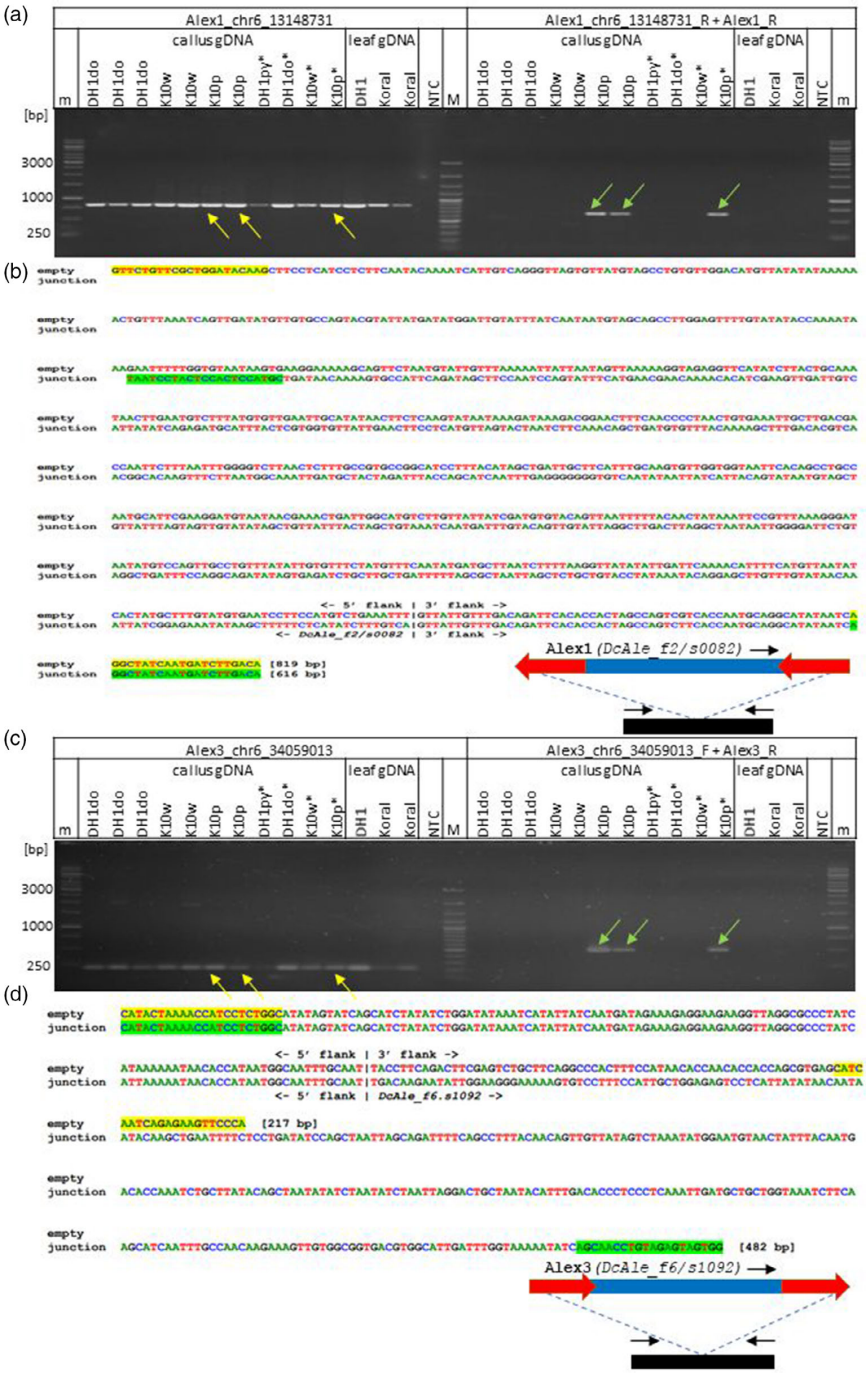
Verification of *de novo* insertions of *Alex1* and *Alex3* in callus. PCR amplification of *Alex1* and *Alex3* insertion sites (a, c) and alignments of the corresponding empty and occupied Sanger‐sequenced sites of *Alex1* and *Alex3* (b, d). Callus samples used for eccDNA sequencing are marked with an asterisk; arrows point to amplicons produced from the empty (yellow) and occupied (green) sites. The expected sizes of the PCR amplicons are shown in parentheses, at the end of each aligned sequence. GeneRuler 1‐kb DNA Ladder (ThermoFisher Scientific) (m) and GeneRuler 100‐bp Plus DNA Ladder (ThermoFisher Scientific) (M) were used as size markers. [Colour figure can be viewed at wileyonlinelibrary.com]

In total, we successfully PCR‐validated one reference insertion site of *Alex1* and *Alex3* each, as positive controls (Figure S18), and eight and seven *de novo* insertion sites of *Alex1* and *Alex3*, respectively (Figures 3 and S19–S22; Tables S19 and S20). As expected, we obtained PCR fragments of sizes representing occupied sites in callus samples from the line in which the insertion was identified using trackposon. The specificity of six PCR fragments was additionally confirmed by Sanger sequencing (Figures 3, S19 and S21; Tables S19 and S20).

#### Validation of *Alex1* and *Alex3* circularization and transcription

**Figure 4:**
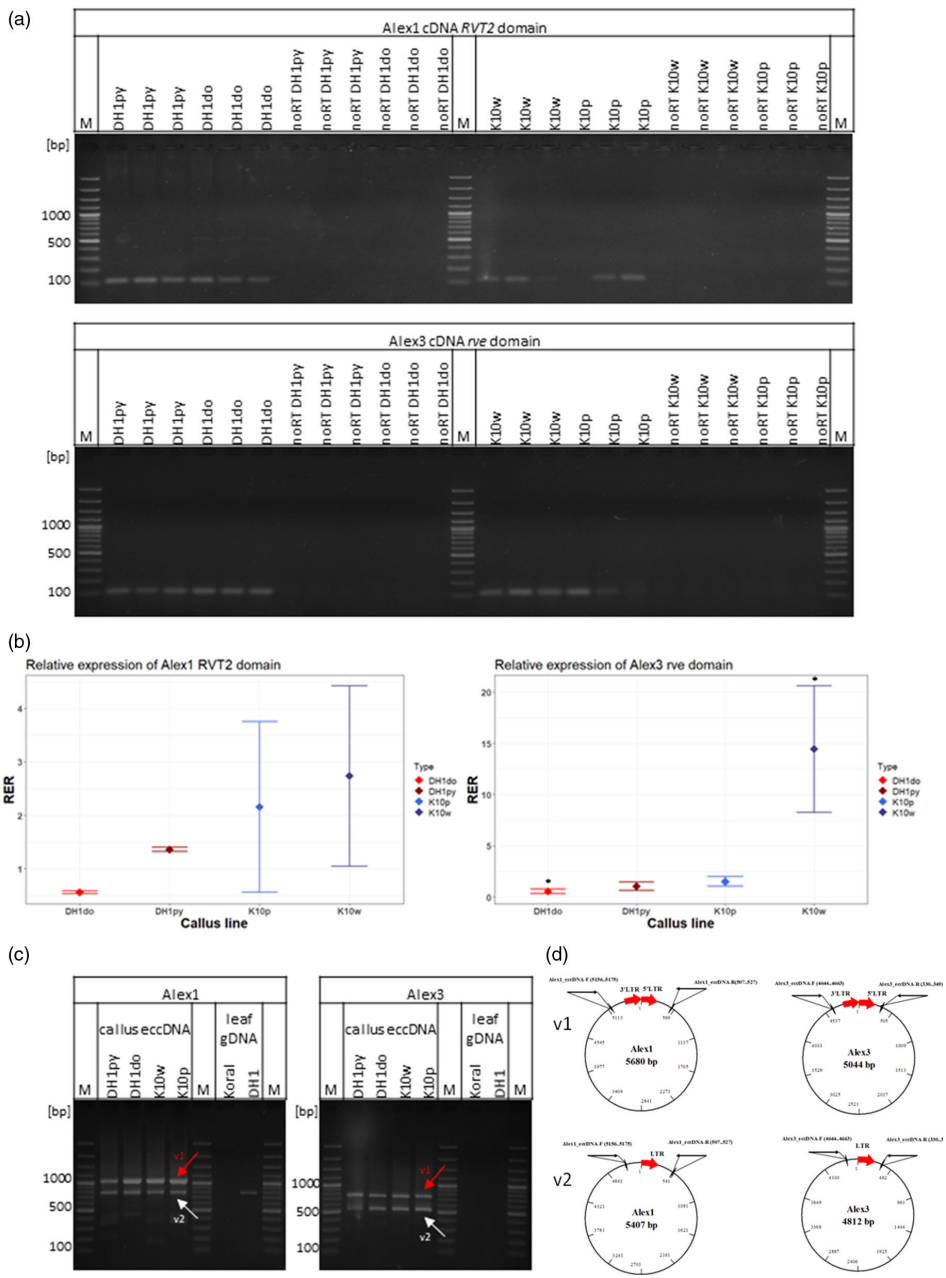
Verification of *Alex1* and *Alex3* transcription and circularization. (a) Results of the RT‐PCR identification of *Alex1* and *Alex3* transcripts. (b) Expression analysis of *Alex1* and *Alex3* domains using RT‐qPCR, (c) PCR amplification of LTR–LTR junctions of circularized *Alex1* and *Alex3*, and (d) schematic representation of eccDNAs. Red arrows in (c)point to amplicons representing LTR–LTR junctions (v1); white arrows point to amplicons representing DNA circles with one LTR (v2); black arrows in (d) indicate the localization of primers. The expected sizes of *Alex1* and *Alex3* LTR–LTR and LTR junctions are 1052 and 779 bp (*Alex1)* and 750 and 518 bp (*Alex3)*, respectively. [Colour figure can be viewed at wileyonlinelibrary.com]

The LTR‐RT‐derived eccDNA may originate from the circularization of linear fragments formed during mobilization or from recombination between the two LTRs. If eccDNA is produced from extrachromosomal DNAs resulting from the mobilization of an active LTR‐RT copy, LTR–LTR junctions are expected to be present. The presence of LTR–LTR junctions in the *Alex1* and *Alex3* eccDNA was confirmed by the identification of raw Illumina reads spanning the ends of both LTRs, separated by small indels at the junction site (Figure S23), and the alignment of reads with the carrot reference genome that match the annotated *Alex1* and *Alex3* LTR‐RTs (Figure S24). We also PCR‐verified the presence of *Alex1* and A*lex3* eccDNAs and transcripts, and quantified the RNA levels of *Alex1* and *Alex3* using reverse‐transcription quantitative PCR (RT‐qPCR) (Figure 4). In the case of both elements, PCR fragments of a size reflecting the presence of two joined LTRs, as well as a single LTR, were detected in eccDNA‐enriched samples (i.e. those used for eccDNA sequencing) but not in the corresponding gDNA samples (Figure 4c). Both *Alex1* and *Alex3* were transcribed in the callus, as confirmed by PCR amplification of its reverse transcriptase (rvt) and integrase (rve) domains (Figure 4a). Although *de novo* insertions of *Alex1* were detected only in the ‘Koral’‐derived callus, its expression was similar in K10 and DH1 callus (Figure 4b). In contrast, *Alex3* was significantly more highly expressed in the K10w subline as compared with the remaining samples (Tukey’s honestly significant difference (HSD), adjusted *P* < 0.05) (Figure 4b). Interestingly, the number of eccDNA reads corresponding to *Alex3* was twofold higher in K10p than in K10w, whereas the number of *de novo* insertions in K10w (19) was higher than in K10p (13).

## DISCUSSION

Transposable elements are important agents driving the evolution of plant genomes. Their ability to amplify in the host genome makes them responsible for substantial differences in plant genome size (Leitch & Leitch, 2013). LTR‐RTs have been shown to affect gene structure and expression (Galindo‐González et al., 2017; Wessler et al., 1995). Moreover, the different histories and dynamics of amplification bursts exhibited by certain LTR‐RT families account for great variation in the structure of even closely related plant genomes.

### LTR‐RT landscape in the carrot genome

Nearly a half of the carrot reference genome has been previously annotated as repetitive DNA, of which retrotransposons (class‐I elements) jointly accounted for two‐thirds of the repetitive fraction. Similar to other plant genomes, the *Copia* (37.7%) and *Gypsy* (21.5%) superfamilies were the most abundant groups (Iorizzo et al., 2016). Our results provided similar estimates; however, intact copies of *Copia* and *Gypsy* superfamilies accounted only for 4.17% and 3.28% of the total carrot genome, whereas the remaining portion represents fragmented or nested copies. Notably, at least 16 Mb (3.36%) of the carrot genome arose from retrotransposition within the last million years. Within carrot LTR‐RTs, we identified lineages common in plant genomes (Neumann et al., 2019; Wicker & Keller, 2007) and showed that carrot LTR‐RTs were represented by a large number of low‐copy‐number subfamilies and only a few more abundant subfamilies.

Plant genomes differ in both the content of repetitive sequences and in their composition. In general, the largest portion of LTR‐RTs contributing to the genome size is usually attributed to families that most recently experienced bursts of activity (El Baidouri & Panaud, 2013), and the genome size correlates well with repeat abundance (Novák et al., 2020a). Unlike in most other plants belonging to the Asterids clade, *Copia* elements were more abundant and diverse, compared with Gypsy elements, in the carrot genome. The prevalence of *Gypsy* elements was reported in *Helianthus* (Qiu & Ungerer, 2018) and in Solanaceae (Aversano et al., 2015; Bolger et al., 2014a; de Assis et al., 2020; Esposito et al., 2019; Gaiero et al., 2019; Leisner et al., 2018; Tomato Genome Consortium, 2012; Xu et al., 2011), whereas a higher proportion of *Copia* was recently reported for *Apium graveolens* (celery) (Song et al., 2020), another representative of the Apiaceae.

The diversity of LTR‐RTs comes from the high error‐prone mechanism of reverse transcription, which may result in new variants of active copies or non‐functional copies (Gabriel et al., 1996). Novel variants may also arise upon recombination between related LTR‐RTs in the course of transposition (Drost & Sanchez, 2019). Also, mutations keep accumulating after the insertion of a new copy into the genome (Wicker & Keller, 2007). All these processes may lead to the inactivation of an LTR‐RT copy or to the establishment of a novel active subfamily. Whereas the former results in the aging and decay of a subfamily, the latter leads to an increase in intra‐lineage/intra‐family LTR‐RT diversity and its proliferation.

Within carrot LTR‐RTs, some lineages such as *DcSIRE* (*Copia*), *DcBianca* (*Copia*), *DcAngela* (*Copia*) or *DcRetand* (*Gypsy*) were characterized by relatively low diversity, as they formed one large cluster grouping all copies. Also, even though those lineages were abundant, the number of subfamilies they comprised was low. In contrast, the most diverse *Copia* lineages, e.g. *DcAle* (*Copia*) and *DcIvana* (*Copia*), despite being less numerous, included many low‐copy‐number or single‐copy subfamilies.

The diversity and abundance of LTR‐RTs was also strongly dependent on their pattern of activity over time. As the LTR‐RT transposition mechanism results in the formation of two identical LTRs flanking the novel copy, the divergence of the LTR sequences has been conventionally applied to estimate the period of activity (Wicker & Keller, 2007). Taking into account the estimated age and number of copies, Wicker & Keller (2007) proposed three patterns of activity of *Copia* elements, reflecting: (i) short periods of intense activity; (ii) permanent activity over longer periods, but at a lower level; and (iii) moderate activity over long time periods, possibly spanning 1–2 Myr. However, the question of how LTR‐RTs evolved to adopt different strategies for their survival in the host genome and which strategy is more beneficial for both the TE and the host remains open (Stritt et al., 2021).

In general, carrot LTR‐RTs were active recently, as indicated by the estimated mean ages of lineages varying from 0.6 to 1.7 Myr. Importantly, 43% of carrot LTR‐RTs have been active within the last million years, with activity peaks within the period of the last 100 thousand years. The age distribution within lineages and the size of subfamilies suggest that *DcSIRE* (*Copia*), *DcAngela* (*Copia*) and *DcRetand* (*Gypsy*) lineages are likely to have expanded within relatively short intense burst(s) of transposition (Figure S25). In contrast, the most diverse lineages, e.g. *DcAle* (*Copia*), *DcIvana* (*Copia*) and *DcReina* (*Gypsy*), grouping mostly low‐copy‐number or single‐copy subfamilies, are likely to have been active continuously at low levels in the carrot genome, and at least some of them were readily mobilized in callus cultures.

The lineages of carrot LTR‐RTs differed in terms of their genomic localization. As expected, they mostly clustered within pericentromeric regions. Intact LTR‐RTs and solo LTRs from the same lineage usually occupied similar positions. The most abundant and less diverse lineages, such as *DcSIRE* (*Copia*) and *DcRetand* (*Gypsy*), were enriched in intergenic regions, whereas copies of elements representing more diverse lineages, in particular *DcAle* (*Copia*) and *DcReina* (*Gypsy*), were more frequently localized near genes. Notably, the genomic localization of copies belonging to both high‐ and low‐copy‐number subfamilies from the same lineage showed similar distributions. It has been reported that *Copia* elements were more frequently associated with genes, compared with *Gypsy* elements (Galindo‐González et al., 2017). However, we were not able to confirm differences in terms of positioning *Copia* and *Gypsy* elements in the carrot genome, possibly because approximately 50 Mb of the 473‐Mb carrot genome is still missing in the current version of the genome assembly. It is likely that the unassembled portion represents mostly the repetitive fraction, rich in LTR‐RT‐derived sequences, as shown for the recent improved plant genome assemblies (Li et al., 2019; Ou et al., 2018).

Low‐copy‐number subfamilies significantly contributed to carrot genome diversity. Nearly half of the annotated LTR‐RTs were grouped into subfamilies comprising fewer than 10 copies. Within low‐copy‐number LTR‐RTs, the *DcAle* lineage is of particular interest, owing to its confirmed mobility (as discussed below). Elements from that lineage are usually grouped into low‐copy‐number families in plant genomes (Esposito et al., 2019; Stritt et al., 2020). In carrot, the DcAle elements show very high levels of intra‐lineage variability, manifested by a large number of low‐copy‐number subfamilies. We speculate that they have been continuously active at a low level, preserving the survival of the lineage with a minimal impact on the integrity of the host genome. Stritt et al. (2020, 2021) suggested that similar characteristics of *Ale* and *Alesia* lineages in other species indeed reflected their evolutionary strategy to persist at low copy numbers.

### LTR‐TRs active in carrot callus cultures

Many reports provided evidence for the activation of LTR‐RTs resulting from physiological stress imposed by tissue cultures (Bayram et al., 2012; Grandbastien et al., 1989; Hirochika, 1993; Hirochika et al., 1996a; Pouteau et al., 1991). eccDNA formation has been proposed as a mechanism preventing LTR‐RT reinsertion and eccDNA sequencing was previously demonstrated to be an efficient tool to search for active retrotransposons (Lanciano et al., 2017). Thus, we used that approach to identify LTR‐RTs mobilized in the callus, in combination with whole‐genome resequencing of the callus lines to mine out newly inserted LTR‐RT copies. A recent analysis showed that the fraction of eccDNAs derived from repeats is proportional to the repeat content in the genome, as eccDNA formation results from random DNA damage (Møller et al., 2020). Similarly, 62% and 23% of Arabidopsis eccDNAs overlapped with genes and TEs, respectively (Wang et al., 2021). Thus, the over‐representation of reads derived from a particular group of LTR‐RTs in the eccDNA library was a good indicator of the actual mobilization. To eliminate the background noise from our eccDNA libraries, we used repeateplorer2‐based reads clustering (Novák et al., 2013; Novák et al., 2020b). The approach was recently demonstrated to be effective (Mann et al., 2021). Finally, it allowed us to identify 10 young low‐copy‐number LTR‐RT subfamilies. Among them, two DcAle subfamilies, confirmed to be active, were represented by the highest numbers of eccDNA reads.

eccDNA can be produced from an extrachromosomal linear LTR‐RT copy via recombination between two LTRs, resulting in the formation of circles containing one LTR, or via non‐homologous end joining (NHEJ) of two LTR ends, resulting in the formation of circles containing LTR–LTR junctions. However, intrachromatid recombination between two LTRs of an inert LTR‐RT would also produce eccDNA containing one LTR (Møller et al., 2015). The presence of short indels at the junction site is a hallmark of NHEJ (Sawyer & Malik, 2006). As for both *Alex1‐* and *Alex3‐*derived eccDNAs, we identified reads supporting the presence of LTR–LTR junctions produced by NHEJ carrying small indels at the junction site, and we conclude that they were generated from extrachromosomal linear copies, confirming the mobilization of *Alex1* and *Alex3*.

All investigated carrot callus sublines produced eccDNAs that could be attributed to different LTR‐RTs. To find elements able to complete the cycle and successfully reinsert into a new genomic position, we identified non‐reference insertion sites using trackposon. False‐positives and false‐negatives affecting the identification of *de novo* insertions were estimated as 5.5% and 2.8%, respectively. Novel copies were observed for two DcAle subfamilies, *Alex1* and *Alex3*, only in the ‘Koral’‐derived K10 callus sublines, whereas in the DH1‐derived callus sublines no novel insertions could be reliably identified, despite the fact that comparable levels of LTR‐RT transcripts and eccDNAs were generally observed in all callus sublines. This suggests that intraspecific differences may exist, influencing the efficiency of LTR‐RT reintegration. Lee et al. (2020) reported that active LTR‐RTs in Arabidopsis, over‐represented in VLPs, were not reinserted but accumulated as eccDNAs.

We have provided evidence for the mobilization of two DcAle subfamilies, *Alex1* and *Alex3*, in the carrot callus, combining information derived from the quantification of expression levels of those elements in the callus, eccDNA sequencing and the identification of LTR‐RT‐derived circles enriched in the pool, the *in silico* identification of novel copies, followed by PCR amplification of LTR–LTR junctions in eccDNA and LTR‐genome junctions from *de novo* insertions in respective genomes. Most *Alex1* and *Alex3* insertion sites were localized in genic regions, consistent with the general characteristics of the *Ale* lineage (Wicker & Keller, 2007). Novel insertions of *Alex1* and *Alex3* were also positioned near genes, which possibly is one of the constituents of the adaptive mechanism allowing *Ale* elements to persist in low copy numbers, probably combined with self‐regulation and the reduced length of internal repeats, as proposed by Stritt et al. (2021). Active LTR‐RTs usually represent stress‐activated low‐copy‐number families, such as *Tos17* (Hirochika et al., 1996a), *Lullaby* (Picault et al., 2009) and *HUO* in rice (Peng et al., 2019), *ONSEN, ATPG* and *ATCOPIA93/Evade* in Arabidopsis (Ito et al., 2011; Lee et al., 2020; Mirouze et al., 2009; Tsukahara et al., 2009), and *Tnt1* and *Tto1* in tobacco (Hirochika et al., 1996b; Pouteau et al., 1991). Active plant LTR‐RTs are often associated with genic regions, e.g. *de novo* insertions of rice *Tos17* were enriched in genes expressed at a moderate level (Yamazaki et al., 2001; Zhang et al., 2020), whereas *VANDAL21* and *ATCOPIA93*, active in Arabidopsis epigenetic recombinant inbred lines (epiRILs) (Mirouze et al., 2009; Tsukahara et al., 2009), were enriched near or within genes (Quadrana et al., 2019). As those elements are often associated with genic regions, the balance between LTR‐RT silencing and mechanisms responsible for the proper maintenance of nearby gene expression may allow their mobilization (Sigman & Slotkin, 2016). Those regulatory mechanisms are likely to be related to the number of LTR‐RT copies. It was shown that DNA methylation increases when additional copies accumulate in the genome, which in turn results in a decrease of transcription and transposition rates (Peng et al., 2019). Thus, both the genic localization and the low copy number are favorable for a given LTR‐RT family to stay active. In addition, stress‐associated hypomethylation of the genome (Viggiano & de Pinto, 2017) often affects LTR‐RT methylation (Atighi et al., 2020; Yu et al., 2013), providing an opportunity for their mobilization. The active carrot *Alex* elements fit well with those observations. The age distribution of the *DcAle* lineage showed one peak less than 0.1 Mya, suggesting constant activity of this lineage and no evident bursts of transposition. Whereas nine copies of *Alex1* were present in the donor plant used to establish the ‘Koral’‐derived callus sublines, a total of 14 new copies were revealed in K10w or K10p, only one copy of *Alex3* was present in the donor and 32 new copies were found in the callus, reflecting the much higher dynamics of *Alex3* in the callus. As proposed by Drost & Sanchez (2019), recombination between two related extrachromosomal copies of LTR‐RTs in the course of transposition may provide an advantage, as it promotes evolvability and facilitates diversification, resulting in the survival of the lineage. The fact that at least two different *DcAle* subfamilies could be activated in the ‘Koral’ genomic background provides means to further investigate their transposition biology and possible interactions between subfamilies.

## EXPERIMENTAL PROCEDURES

### Plant material and genomic data

The carrot DH1 reference genome (GenBank assembly accession no. GCA_001625215.1; Iorizzo et al., 2016) was used to search for LTR‐RTs.

Each of the two callus lines derived from the DH1 line and ‘Koral’ cv. (DH1 and K10, respectively) were divided into pairs of sublines differing in callus morphology (Figure S8). The DH1‐derived sublines were pale yellow (DH1py) and dark orange (DH1do) (Klimek‐Chodacka et al., 2018), whereas those derived from ‘Koral’ were white (K10w) and purple (K10p) (Oleszkiewicz et al., 2018). The callus sublines were kindly provided by Prof. Rafał Barański (University of Agriculture in Krakow, Poland) and used to investigate the activity of LTR‐RTs.

Raw reads from 31 resequenced genomes of *D. carota* (NCBI Sequence Read Archive, accession SRP062070, under umbrella project PRJNA285926; Table S18), comprising 13 wild and 18 cultivated carrot accessions (Iorizzo et al., 2016) were used to search for insertion sites of LTR‐RTs identified as active in callus cultures.

### Mining and classification of LTR retrotransposons in the carrot genome

The LTR‐RTs were identified and clustered using an approach described by El Baidouri & Panaud (2013). In brief, LTR‐RTs were retrieved *de novo* with ltrharvest (Ellinghaus et al., 2008) using ‐seed 80 ‐maxlenltr 4000 ‐mindistltr 3000 ‐mintsd 2 ‐maxtsd 20 ‐motif tgca, with other parameters set as default. Clustering into subfamilies was performed for LTR sequences with silix 1.2.9 (Miele et al., 2011) using parameters ‐‐ident 0.6 ‐‐overlap 0.7 for classification into a subfamily. We further analyzed all subfamilies and single‐copy elements (i.e. those not classified to a subfamily based on the clustering results). Conserved domains characteristic for plant LTR‐RT lineages were identified in each copy using repeatexplorer dante and filtered using default settings of the dante ‘Protein Domains Filter Tool’ (Novák et al., 2010; Novák et al., 2013, Novák et al. 2020) and classified according to Neumann et al. (2019). Subfamilies were classified into lineages if at least one LTR‐RT domain was identified in at least one copy representing a subfamily.

Families were defined based on the reverse transcriptase (*rt*) domain phylogeny. Nucleotide sequences of *rt* domains were aligned using mafft 7.471 (Katoh & Standley, 2013) with the following parameters: ‐‐localpair ‐‐maxiterate 1000. Gaps were removed from the alignment using gblocks 0.91b (Talavera & Castresana, 2007). Trees were inferred in mega 6 (Tamura et al., 2013) based on the general time reversible model (Nei & Kumar, 2000). Initial tree(s) for the heuristic search were obtained by applying the neighbor‐joining method to a matrix of pairwise distances estimated using the maximum composite likelihood (MCL) approach. A discrete Gamma distribution was used to model evolutionary rate differences among sites (four categories; +G, parameter = 2.0763). All positions with less than 90% site coverage were discarded. That is, fewer than 10% alignment gaps, missing data and ambiguous bases were allowed at any position. Branch support was estimated using 1000 bootstrap replicates. The following criteria were used to define families: (i) the branch was supported by a bootstrap of higher than 70; and (ii) the genetic distance to other groups, reflected by the branch length, was at least 0.3. The families were labeled consecutively from ‘f1’ to ‘fn’, whereas truncated elements not grouping with any complete copy were combined into one artificial family per lineage and labeled ‘f0’. LTR‐RTs reported by ltrharvest were renamed according to the following rule: superfamily_*Dc* (stands for *Daucus carota*); lineage_family (as defined by the phylogenetic analysis of ‘*rt*’); silix‐based subfamily_genomic localization.

The age of each copy was estimated based on the similarity of its LTRs. Left and right LTRs were aligned using mafft 7.471 (Katoh & Standley, 2013), with default parameters. DNA distance was calculated in r ape 5.4–1 (Paradis & Schliep, 2019) using the ‘K80’ model. Subsequently, insertion time was estimated using the formula *T* = *K*/2*r*, with the substitution rate of 1.3 × 10^−8^ per site per year, as proposed by Ma et al. (2004) and Wicker & Keller (2007).

To define genomic positions of solo LTRs, we used sequences of LTRs extracted from full‐length LTR‐RTs as a query for a blastn (blastall 2.2.26; Altschul et al., 1997) against the carrot reference genome disabling sequence filtering with Dust (‐F F), with other settings set as default. Using a custom perl script, based on the bioperl module (Stajich et al., 2002), we parsed the blast output file to report hits showing a minimum 80% similarity and 90% coverage. The obtained bed file was compared with the genomic localization of intact LTR‐RTs using bedtools 2.26.0 (Quinlan & Hall, 2010) and LTRs not overlapping with intact copies were retrieved.

Genomic positions of all copies of intact LTR‐RTs and solo LTRs were determined based on the National Center for Biotechnology Information (NCBI) carrot genome annotation (GCF_001625215.1_ASM162521v1_genomic.gff) using bedtools 2.26.0 (Quinlan & Hall, 2010), as described by Macko‐Podgórni et al. (2019), with upstream and downstream regions of 1 kb from the nearest gene. To compare the genomic distribution of intact copies of *Copia* and *Gypsy* elements, we calculated distances from the nearest gene (from 1 to 10 000 bp), divided them into 1‐kb bins, counted the number of *Copia* and *Gypsy* elements in the bins and tested the distribution using the χ^2^ goodness‐of‐fit test in r 4.0.0 (R Core Team, 2020).

### Identification of eccDNA corresponding to LTR retrotransposons mobilized in callus cultures

We sequenced and analyzed eccDNA using the approach described by Lanciano et al. (2017, 2021). In brief, total DNA was extracted using a modified cetyltrimethylammonium bromide (CTAB) method (Briard et al., 2000). The DNA samples were enriched with the eccDNA fraction and libraries for mobilome sequencing were prepared according to the protocol described by Lanciano et al. (2017, 2021). Following read quality control, short reads obtained from paired‐end Illumina sequencing were mapped against carrot chloroplast (GeneBank ID: DQ898156.1) and mitochondrial (GeneBank ID: JQ248574.1) genomes to remove reads originating from organellar genomes.

The eccDNA reads were used for repeatexplorer (Novák et al., 2010; Novák et al., 2013, Novák et al. 2020) to perform comparative analysis with the use of the curated carrot LTR‐RT database. Analysis was run for a random sample of 500 000 reads, equally representing eccDNA reads of the two pairs of callus sublines, using a minimum cluster size for assembly = 3 and default settings for the other parameters. Based on the annotation, we kept clusters meeting the following criteria: automatic annotation as class‐I TE; over 50% reads with hits to the carrot LTR‐RT database; and cluster size > 500. We also performed pairwise χ^2^ test for retrieved clusters, separately for DH1 and K10 callus sublines, between the total number of reads used for comparative analysis and the number of reads grouped in the cluster.

Additionally, to obtain the complete sequence of the LTR‐RT, the *de novo* assembly of eccDNA raw reads was carried out using the a5‐miseq pipeline (Coil et al., 2015). blastn (blastall 2.2.26, Altschul et al., 1997) against the carrot LTR‐RT database was used to identify eccDNA contigs representing LTR‐RTs. Contigs aligned with 90% sequence identity and 80% length coverage were selected for closer inspection.

Subsequently, the mapping of eccDNA reads against the carrot LTR‐RT database using bowtie2 2.3.2 (Langmead & Salzberg, 2012) was performed, with default parameters. The number of mapping reads was normalized by calculating reads per kilo base per million mapped reads (RPKM) values.

### Identification of LTR retrotransposons neo‐insertions in callus cultures

Genomic DNA of three callus sublines (DH1do, K10w and K10p) was extracted using a modified CTAB method (Briard et al., 2000) and purified using NucleoSpin® gDNA Clean‐up (Macherey‐Nagel). Subsequently, DNA Insert Size Libraries of 200–800 bp were constructed and used for PE150 paired‐end sequencing on Illumina HiSeq X (Genomed SA, http://www.genomed.pl).

Raw reads of callus sublines, 30 resequenced carrot genomes and DH1 were pre‐processed using trimmomatic 0.35 (Bolger et al., 2014b), with parameters minqual = 28, minlen = 50, LEADING:28, TRAILING:28, SLIDINGWINDOW:10:28 and MINLEN:50, with quality controlled using fastqc (Andrews, 2010), and reads were mapped to the DH1 genome (Iorizzo et al., 2016; GCA_001625215.1) using bwa‐mem 0.7.12 (Li & Durbin, 2009). The number of mapped reads was used for a rough estimation of the genome coverage using the formula: coverage = (mapped read count * read length)/total genome size.

To identify *de novo* insertions, raw reads produced for the callus sublines were analyzed with trackposon (Carpentier et al., 2019). Reads from the DH1 reference genome were included in the analysis as a control. We set trackposon to report insertion sites supported by at least two reads to allow the detection of low‐frequency *de novo* somatic insertions in the heterogenic callus, likely comprising different cell lineages arising over the course of the culture.

To avoid an increase in false positives, we added a step allowing the identification of soft‐clipped reads to the trackposon pipeline: (i) mapping reads aligned with LTR‐RTs and their mate pairs against the reference genome using bowtie2 2.3.2 (Langmead & Salzberg, 2012), with parameters ‐‐time ‐‐very‐fast‐local; (ii) extracting soft‐clipped reads using SE‐MEi extractSoftclipped (https://github.com/dpryan79/SE‐MEI); and (iii) aligning them back to the reference genome using blastn (blast+ 2.6.0). Finally, those reads were extracted using a script included in the trackposon pipeline (Carpentier et al., 2019). It allowed the fast and efficient identification of specific, non‐reference insertion sites in regions comprising up to 200 bp. For defined genomic positions of new insertions, we designed site‐specific primers and used them for PCR‐based validation.

Insertion sites of RTs active in tissue cultures were also identified in 30 resequenced carrot genomes (Table S10, Iorizzo et al., 2016) using trackposon, with default parameters (Carpentier et al., 2019).

To validate the performance of the modified pipeline we masked the reference insertion sites of *Alex1* and *Alex3* in the carrot DH1 reference genome (Iorizzo et al., 2016) and ran the pipeline using reads of the three callus sublines (DH1do, K10w and K10p). Reads used to construct the reference genome (from Iorizzo et al., 2016) were used as a control. The assumption was that the reference insertions should not be identified in the non‐masked genome, whereas they should be identified in the masked genome.

### Validation of mobility and reintegration of LTR retrotransposons

To verify the presence of LTR‐RTs in the eccDNA fraction, PCR primer pairs were anchored in internal parts of respective LTR‐RTs and directed towards the termini of the element (Table S21) to confirm circularization. DNA samples enriched with the eccDNA fraction, diluted 1000×, were used as templates for PCR.

To verify the presence of transcripts of mobilized LTR‐RTs, PCR on cDNA templates was carried out. Total RNA was extracted from the two pairs of carrot callus sublines (DH1py and DH1do; K10w and K10p) in three replicates each, using NucleoSpin® RNA Plant and Fungi kit (Macherey‐Nagel) following the manufacturer’s protocol. Subsequently, RNA was purified and reverse‐transcribed as described by Macko‐Podgórni et al. (2017). PCR was performed with a primer pair anchored in the integrase (*rve*) and reverse transcriptase (*RVT2*) domains of respective LTR‐RTs (Table S22). As a negative control, RNA samples with no reverse transcriptase (noRT) were used.

RT‐qPCR was performed using quantstudio 3 (Applied Biosystems, now ThermoFisher Scientific). GADPH (LOC108223758) was used as a reference gene. Primer efficiencies were calculated as described by Bowman et al. (2014). Relative expression ratios (RERs) were calculated using the ΔΔ*C*_t_ method (Livak & Schmittgen, 2001). Statistical analysis including analysis of variance (ANOVA) and Tukey’s HSD post‐hoc tests (Tukey, 1949) were performed in r 4.0.0 (R Core Team, 2020).

Novel insertion sites of *Alex1* and *Alex3* were validated by PCR and Sanger sequencing. PCR was carried out with pairs of primers flanking the insertion sites and a primer anchored in the internal portion of *Alex1* (Table S23) or *Alex3* (Table S24), respectively.

Primers used for molecular analyses were designed using primer‐blast (Ye et al., 2012) and verified with oligoanalyzer (IDT, https://www.idtdna.com). The PCR mix included: primers, 10 μm of each; 25 mm of dNTPs (ThermoFisher Scientific); 0.5 U DreamTaq DNA Polymerase (ThermoFisher Scientific); 1× DreamTaq™ Green Buffer (ThermoFisher Scientific); and the DNA or cDNA template. PCR amplifications were performed using the following thermal conditions: 94°C (2 min), 30 cycles of 95°C (30 s), 56°C/58°C (30 s), 68°C (30 s) and 68°C (5 min). The amplicons were separated in 1% agarose gels and stained with MidoriGreen (Nippon Genetics). The products were purified from the gel, cloned in *Escherichia coli* strain DH10B and Sanger sequenced (Genomed SA). The resulting sequences were aligned and analyzed with bioedit (Hall, 1999).

### Statistics and plots

All statistical tests were calculated in r 4.0.0 (R Core Team, 2020). For data sets containing values below 5, simulated *P*‐values were used for calculation. Charts were plotted in r 4.0.0 using the following packages: ggplot2 (https://github.com/tidyverse/ggplot2/issues), sunburstr 2.1.3 (https://github.com/timelyportfolio/sunburstR), biocircos 0.3.4 (https://github.com/lvulliard/BioCircos.R), corrplot 0.84 (https://github.com/taiyun/corrplot), performanceanalytics 2.0.4 (https://github.com/braverock/PerformanceAnalytics) and corrr 0.4.2 (https://github.com/tidymodels/corrr).

## CONFLICT OF INTEREST

The authors declare that they have no conflicts of interest associated with this work.

## AUTHOR CONTRIBUTIONS

DG and AMP conceived and supervised the project. KK, DG and AMP designed the experiments. KK, PK, MH and AMP performed the experiments. KK and AMP analyzed the data. KK, DG and AMP wrote the article. MM and OP helped in analyzing the data and writing the article. AMP, MM and OP acquired the funding. All the authors read and approved the final version for publication.

## Supporting information


**Figure S1.** Distribution of the estimated insertion times for families belonging to *Copia* lineages, containing at least two families with at least 10 copies per family.
**Figure S2.** Maximum‐likelihood trees of *Gypsy* elements.
**Figure S3.** Sunburst chart for low (≤10) and high (>10) copy number subfamilies (inner ring), their localization in the genome (middle ring), and their classification into families (outer ring).
**Figure S4.** Visualization of correlations among features characterizing carrot LTR‐RT lineages, Pearson correlation coefficients with *P*‐value significance codes (***P < 0.001, **P < 0.01, *P < 0.05, *P* < 0.1), histograms with kernel density and scatter plots with fitted lines.
**Figure S5.** Mean ages and confidence intervals for *Copia* and *Gypsy*, calculated for subfamilies containing more than 10 elements.
**Figure S6.** Distribution of genes (I), intact *Copia* (II), *Copia* solo LTRs (III), intact *Gypsy* (IV) and *Gypsy* solo LTRs (V) on the carrot chromosomes.
**Figure S7.** Genomic localization of carrot LTR‐TRs and solo LTRs.
**Figure S8.** The origin of K10 (Klimek‐Chodacka et al., 2018) and DH1 (Oleszkiewicz et al., 2018) callus sublines used for eccDNA sequencing.
**Figure S9.** Graphical representation of clusters attributed to *Alex1* and *Alex2*.
**Figure S10.** Localization of *Alex* copies on carrot chromosome 1 and their distribution in cultivated and wild carrots.
**Figure S11.** Localization of *Alex* copies on carrot chromosome 2 and their distribution in cultivated and wild carrots.
**Figure S12.** Localization of *Alex* copies on carrot chromosome 4 and their distribution in cultivated and wild carrots.
**Figure S13.** Localization of *Alex* copies on carrot chromosome 5 and their distribution in cultivated and wild carrots.
**Figure S14.** Localization of *Alex* copies on carrot chromosome 6 and their distribution in cultivated and wild carrots.
**Figure S15.** Localization of Alex copies on carrot chromosome 7 and their distribution in cultivated and wild carrots.
**Figure S16.** Localization of *Alex* copies on carrot chromosome 8 and their distribution in cultivated and wild carrots.
**Figure S17.** Localization of *Alex* copies on carrot chromosome 9 and their distribution in cultivated and wild carrots.
**Figure S18.** Reference insertions of *Alex1* and *Alex3*.
**Figure S19.** PCR verification of *de novo* insertion site of *Alex1*, confirmed by Sanger sequencing.
**Figure S20.** Amplification of *de novo* insertion sites of *Alex1*.
**Figure S21.** PCR verification of *de novo* insertion site of *Alex3*, confirmed by Sanger sequencing.
**Figure S22.** Amplification of *de novo* insertion sites of *Alex3*.
**Figure S23.** Variants of LTR–LTR junction reads identified in the K10p eccDNA library.
**Figure S24.** IGV view of alignments of eccDNA reads derived from K10w and K10p callus sublines to copies of *Alex1* and *Alex3* in the carrot reference genome DH1.
**Figure S25.** Krakow density plots showing the age distribution of carrot LTR‐RTs, representing the eight most numerous subfamilies (containing more than 100 copies).

**Figure S3.** Sunburst chart for low‐ (≤10) and high (>10) copy number subfamilies (inner ring), their localization in the genome (middle ring), and their classification into families (outer ring). More details are provided in the html version of the figure (Figure_S3.html).

**Table S1.** Superfamilies of LTR‐RTs identified in the carrot DH1 reference genome.
**Table S2.** Characteristics of LTR‐RT lineages in the carrot DH1 reference genome.
**Table S3.** Mapping statistics of eccDNA reads to the carrot DH1 reference genome.
**Table S4.** Statistics of eccDNA read assemblies.
**Table S5.** Summary of repeatexplorer clusters annotation.
**Table S6.** Characteristics of LTR‐RTs overrepresented in mobilomes of four carrot callus sublines lines identified based on the repeatexplorer comparative analysis.
**Table S7.** Number and proportion of reads attributed to each carrot LTR‐RT after the merging of clusters representing individual elements.
**Table S8.** Characterization of LTR‐RT superfamilies identified in eccDNA, including clusters representing each superfamily, domains detected by repeatexplorer , age and abundance of superfamily based on copies in the reference genome.
**Table S9.** Summary statistics of callus subline sample sequencing results.
**Table S10.** Validation of the performance of the modified trackposon pipeline.
**Table S11.** Summary of *de novo* insertion sites identified for LTR‐RT enriched in eccDNA.
**Table S12.** Insertion sites of *Alex1* in K10p and K10w callus sublines.
**Table S13.** Insertion sites of *Alex2* in K10w and K10p callus sublines.
**Table S14.** Insertion sites of *Alex3* in K10w and K10p callus sublines.
**Table S15.** Insertion sites of *Ivan1* in K10w and K10p callus sublines.
**Table S16.** Insertion sites of *DcTork_f0/s1917* in K10w and K10p callus lines.
**Table S17.** Insertion sites of *DcTork_f1/s2099* in K10w and K10p callus sublines.
**Table S18.** List of *Daucus carota* accessions with resequenced genomes.
**Table S19.** Summary of the verification of *de novo* insertion sites of *Alex1* in K10p and K10w callus sublines.
**Table S20.** Summary of the verification of *de novo* insertion sites of *Alex3* in K10w and K10p callus sublines.
**Table S21.** Primers for verification of LTR‐RT circularization.
**Table S22.** Primers for verification of the presence of the LTR‐RTs domain transcripts and for RT‐qPCR analysis.
**Table S23.** Primers for verification of *de novo* insertions of *Alex1*.
**Table S24.** Primers used for verification of *de novo* insertions of *Alex3*.

**Data S1.** Age of carrot LTR‐RTs.

**Data S2.** Correlations among features characterizing carrot LTR‐RT lineages.

**Data S3.** Alignment of *Alex1* DH1 reference copies with 20‐bp flanking region and eccDNA‐derived contig with annotation of LTRs, PBS, PPT and TSD.

**Data S4.** Alignment of *Alex3* DH1 reference copy with 20‐bp flanking region and eccDNA‐derived contig with annotation of LTRs, PBS, PPT and TSD.

**Data S5.** Insertion sites of *Alex1*, *Alex2* and *Alex3* in cultivated and wild carrots.

## Data Availability

Raw sequencing data are available at NCBI BioProjects (WGS sequencing:PRJNA708189; mobilome/eccDNA sequencing:PRJNA712991).
