# Supplementary material for: Diverse and mobile: eccDNA‐based identification of carrot low‐copy‐number LTR retrotransposons active in callus cultures
Source: Plant J. 2022 May 10;110(6):1811–28. doi: 10.1111/tpj.15773 (PMC9324142; doi:10.1111/tpj.15773)
Supplement: Supplementary file 1 — Figure S1. Distribution of the estimated insertion times for families belonging to Copia lineages, containing at least two families with at least 10 copies per family. Figure S2. Maximum‐likelihood trees of Gypsy elements. Figure S3. Sunburst chart for low (≤10) and high (>10) copy number subfamilies (inner ring), their localization in the genome (middle ring), and their classification into families (outer ring). Figure S4. Visualization of correlations among features characterizing carrot LTR‐RT lineages, Pearson correlation coefficients with P‐value significance codes (***P < 0.001, **P < 0.01, *P < 0.05, P < 0.1), histograms with kernel density and scatter plots with fitted lines. Figure S5. Mean ages and confidence intervals for Copia and Gypsy, calculated for subfamilies containing more than 10 elements. Figure S6. Distribution of genes (I), intact Copia (II), Copia solo LTRs (III), intact Gypsy (IV) and Gypsy solo LTRs (V) on the carrot chromosomes. Figure S7. Genomic localization of carrot LTR‐TRs and solo LTRs. Figure S8. The origin of K10 (Klimek‐Chodacka et al., 2018) and DH1 (Oleszkiewicz et al., 2018) callus sublines used for eccDNA sequencing. Figure S9. Graphical representation of clusters attributed to Alex1 and Alex2. Figure S10. Localization of Alex copies on carrot chromosome 1 and their distribution in cultivated and wild carrots. Figure S11. Localization of Alex copies on carrot chromosome 2 and their distribution in cultivated and wild carrots. Figure S12. Localization of Alex copies on carrot chromosome 4 and their distribution in cultivated and wild carrots. Figure S13. Localization of Alex copies on carrot chromosome 5 and their distribution in cultivated and wild carrots. Figure S14. Localization of Alex copies on carrot chromosome 6 and their distribution in cultivated and wild carrots. Figure S15. Localization of Alex copies on carrot chromosome 7 and their distribution in cultivated and wild carrots. Figure S16. Localization of Alex copie [file TPJ-110-1811-s008.pdf]

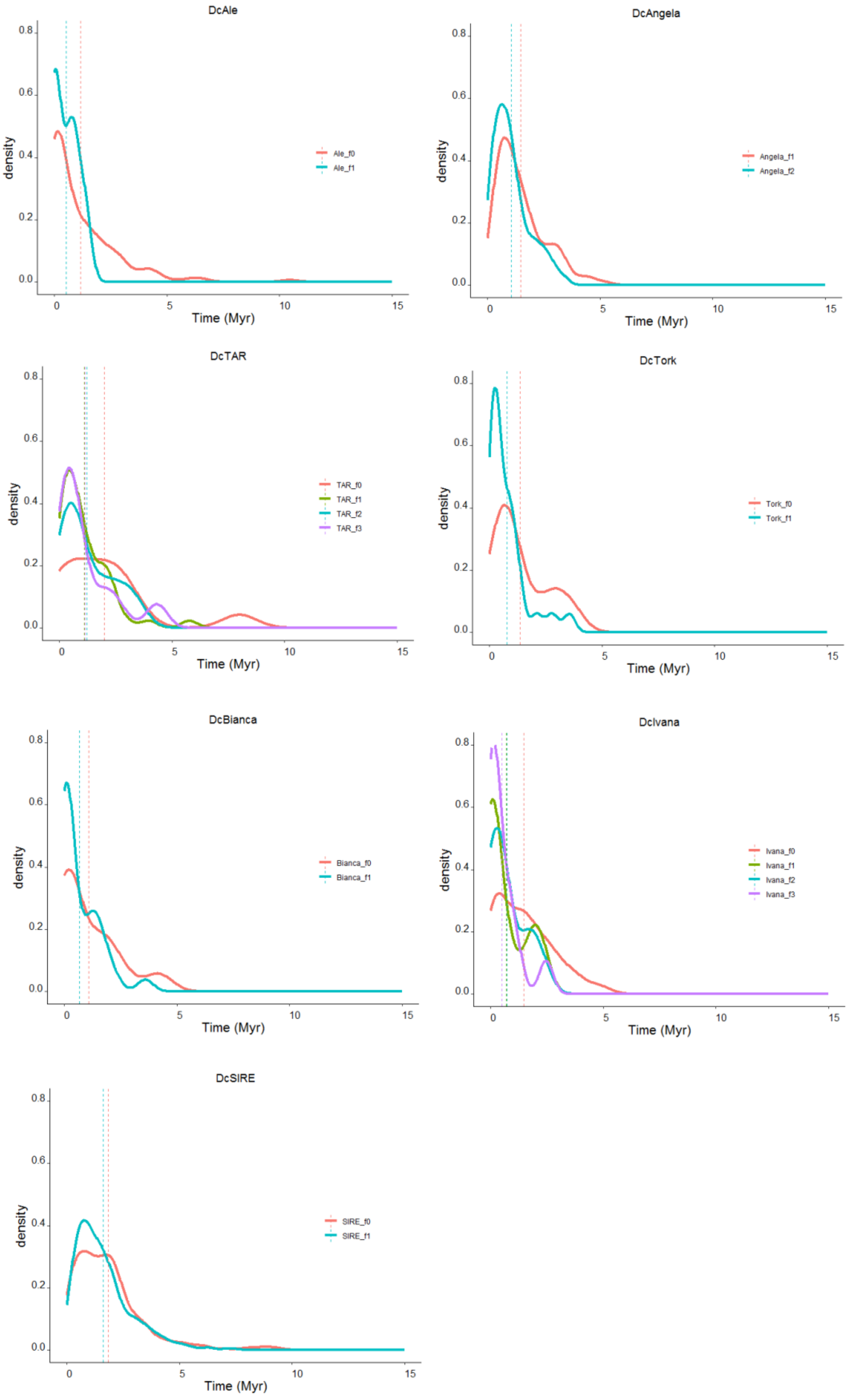

Figure S1. Distribution of estimated insertion times for families belonging to *Copia* lineages, containing at least two families with at least 10 copies per family. Average insertion times per family are indicated by dashed lines.

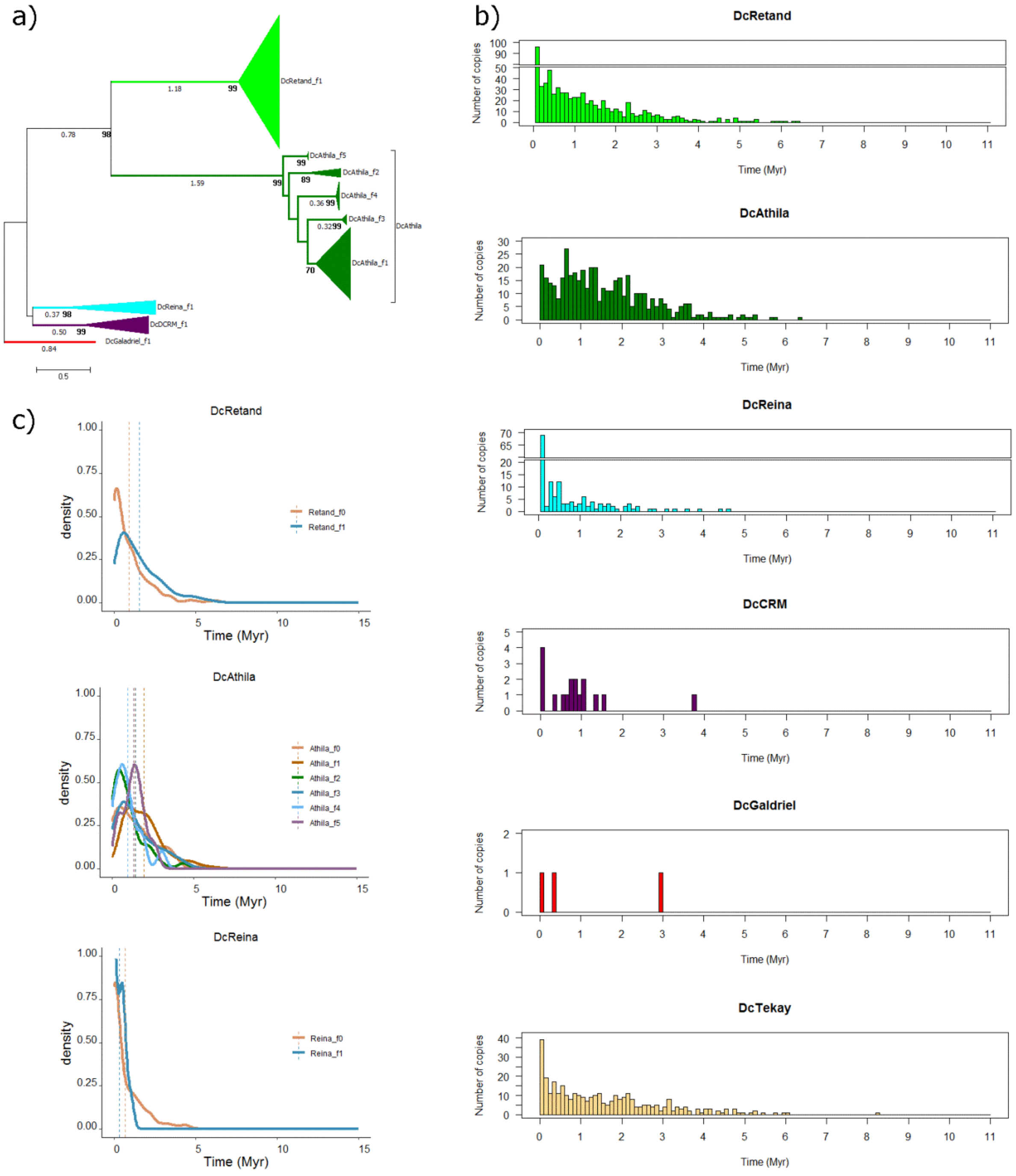

Figure S2. Maximum Likelihood tree of *Gypsy* elements, based on RT domains of 216 copies (a) distribution of insertion time (in 0.1 Myr bins) of all elements belonging to *Gypsy* lineages (b) and distribution of insertion time within families for *Gypsy* lineages containing at least two families with at least 10 copies per family (c). Branch length (numbers below branches) represent the number of substitutions per site. Branch support was estimated with 1000 bootstrap replicates (numbers below branches, in bold). Average insertion times per family are indicated by dashed lines.

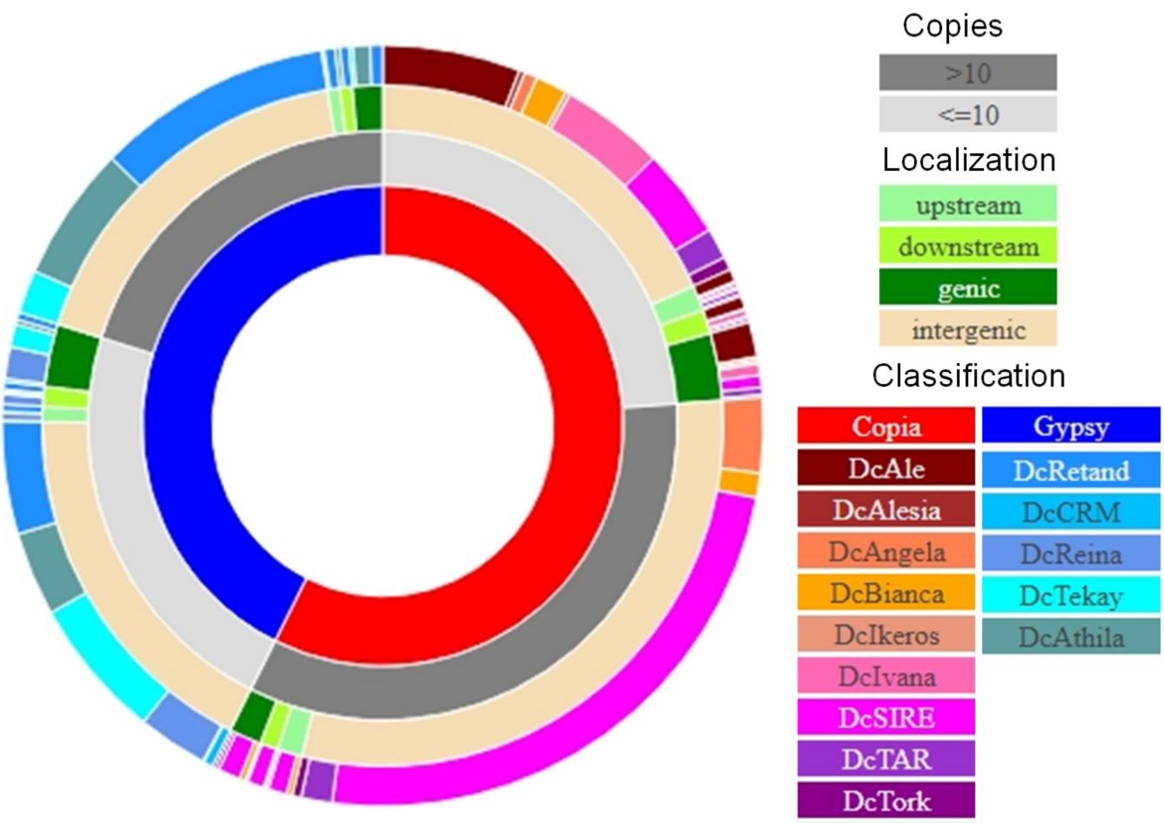

Figure S3. Sunburst chart for low- (<=10) and high (>10) copy number subfamilies (inner ring), their localization in the genome (middle ring), and their classification into families (outer ring). More details are provided in the html version of the figure (**Figure_S3.html**).

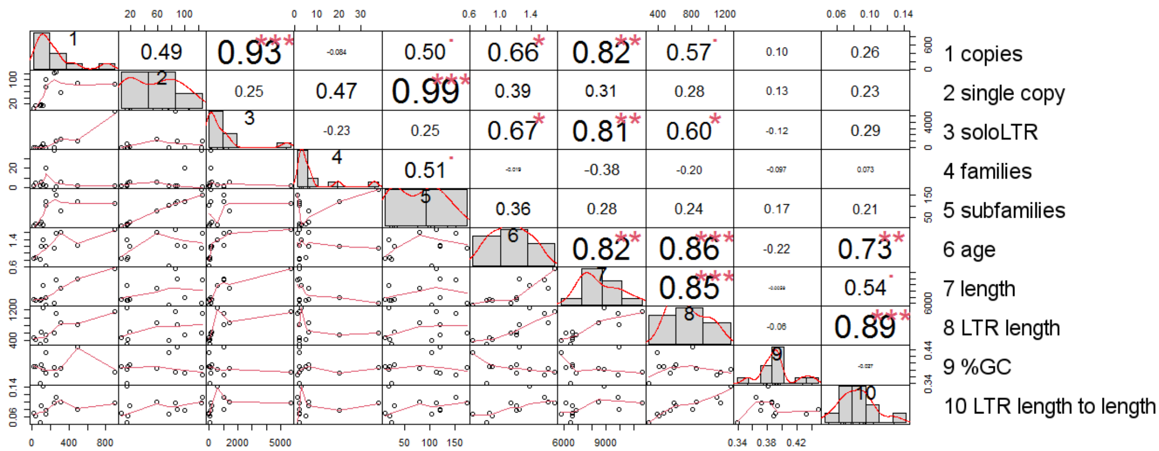

Figure S4. Visualization of correlations among features characterizing carrot LTR-RT lineages, Pearson correlation coefficients with p-value significance codes (<0.001***, 0.001=**, 0.01=*, 0.05 ·), histograms with kernel density, and scatter plots with fitted lines.

a)

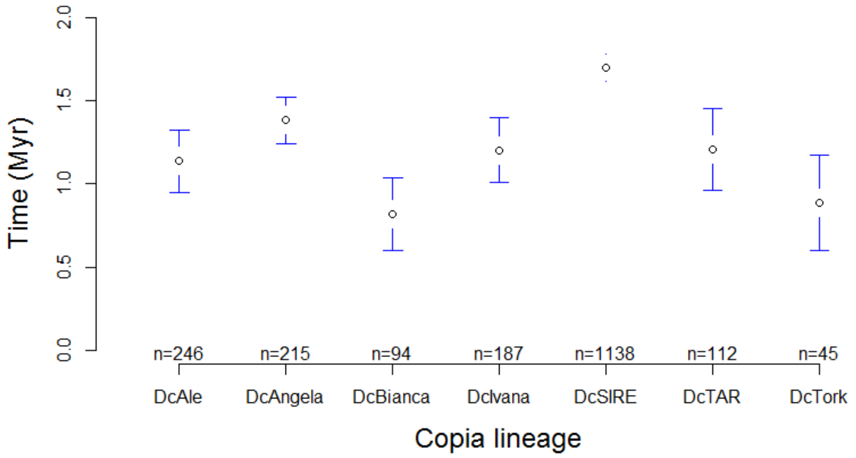

b)

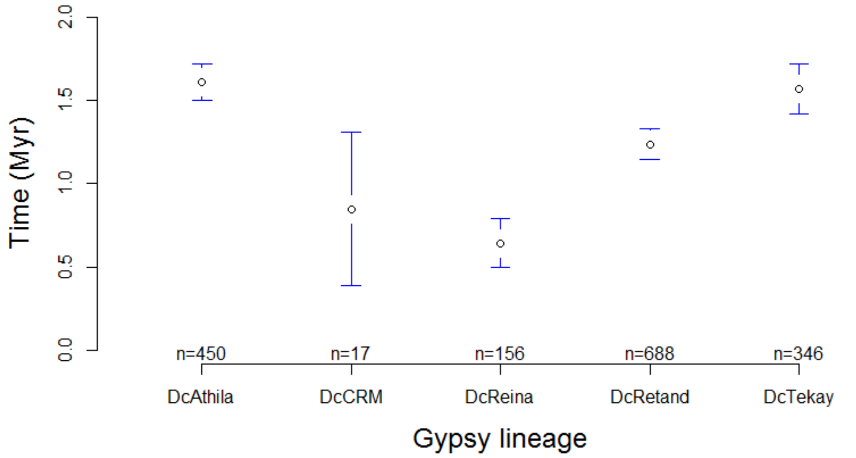

Figure S5. Mean ages and confidence intervals for *Copia* (a) and *Gypsy* (b), calculated for subfamilies containing more than 10 elements.

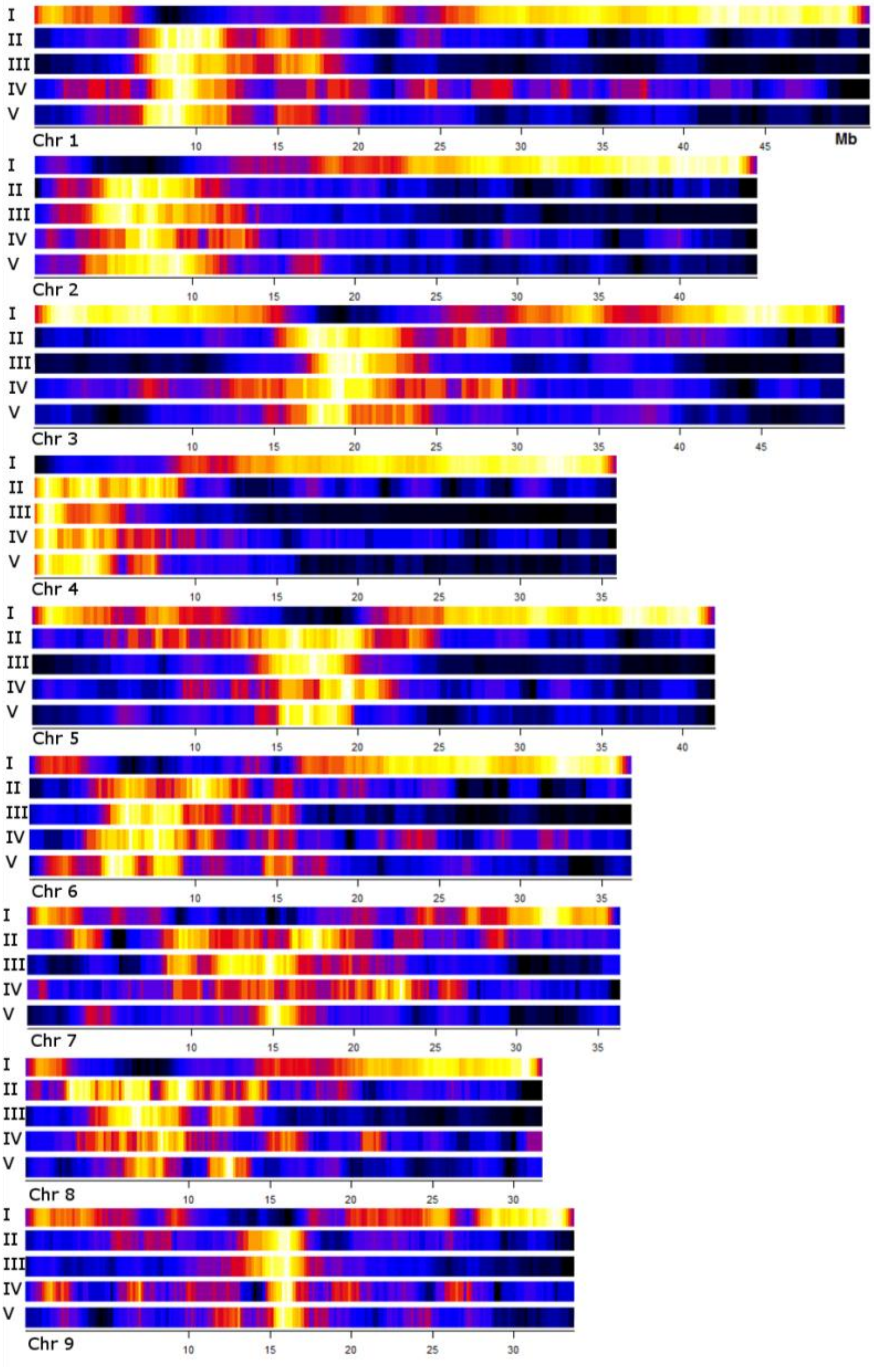

Figure S6. Distribution of genes (I), intact *Copia* (II), *Copia* soloLTRs (III), intact *Gypsy* (IV), and *Gypsy* soloLTRs (V) on the carrot chromosomes.

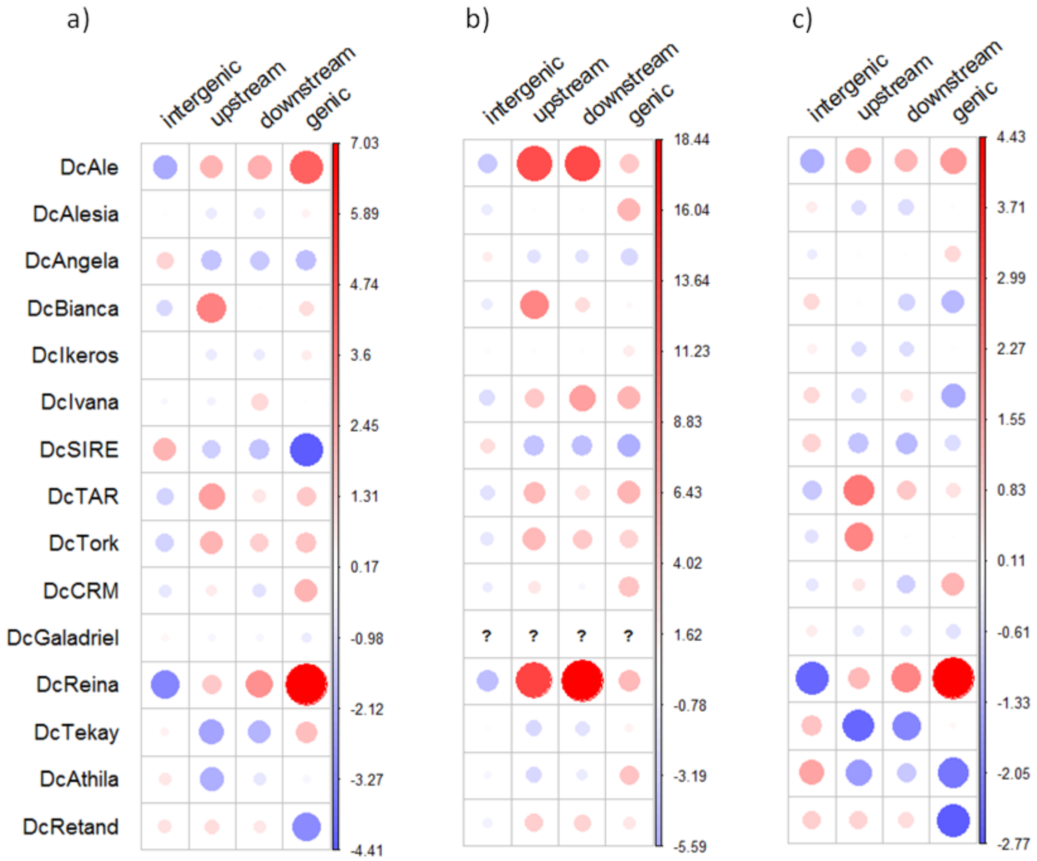

Figure S7. Genomic localization of carrot LTR-TRs and soloLTRs. Differences in the distribution of (a) all elements belonging to different lineages ($p = 5e\text{-}4$), (b) soloLTRs ($p = 5e\text{-}4$), and (c) elements representing subfamilies containing less than 10 copies ($p = 9e\text{-}4$) in the DH1 carrot reference genome. Color scale reflects deviation from the average value. The size of circles is proportional to the contribution of each test to the total Pearson chi-squared score, and the question marks represent lineages with zero values.

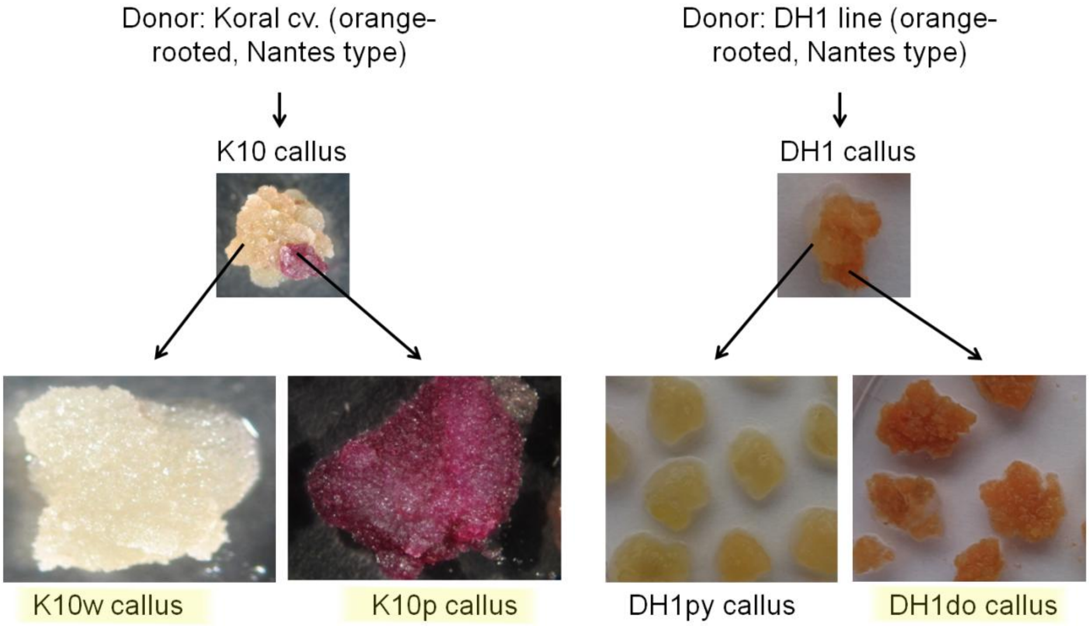

Figure S8. The origin of K10 (Klimek-Chodacka el al. 2018) and DH1 (Oleszkiewicz et al. 2018) callus sub-lines used for eccDNA sequencing. Names of callus sub-lines used for whole genome resequencing are highlighted in yellow.

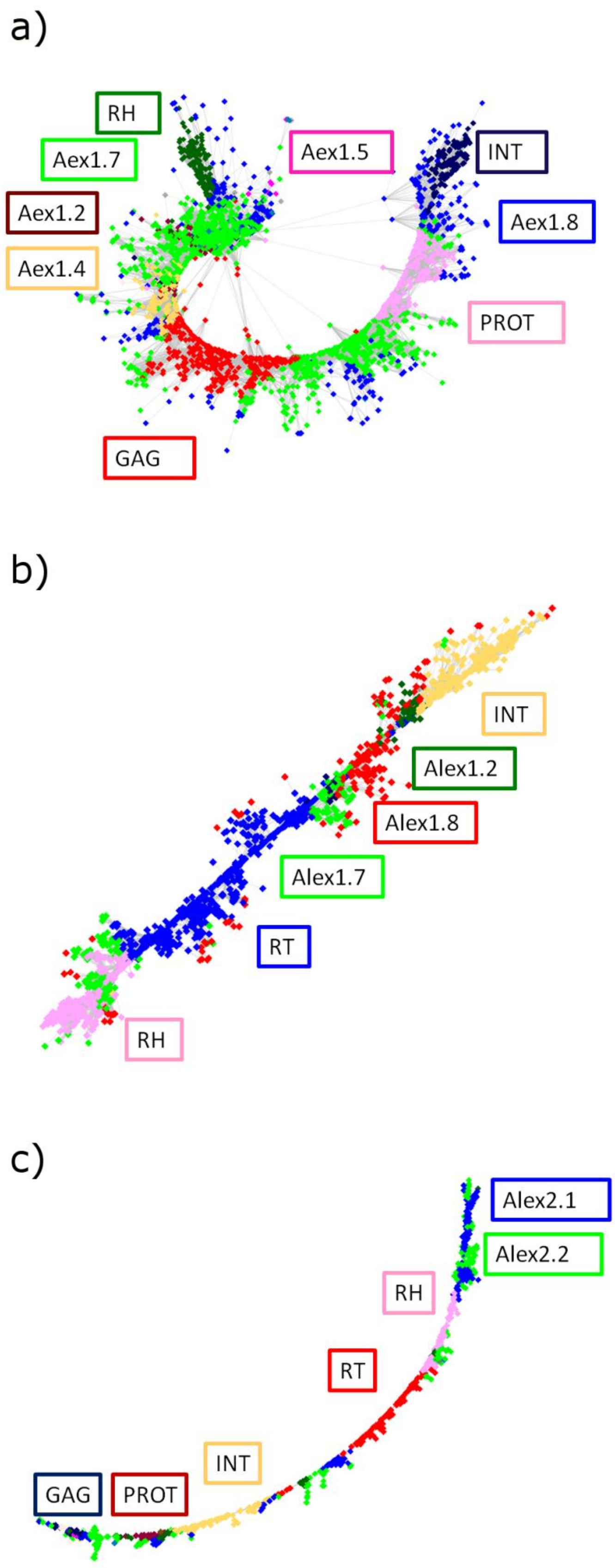

Figure S9. Graphical representation of clusters attributed to *Alex1* (a, b) and *Alex2* (c). Dots representing individual reads are colored based on similarity hits to domains of *Ale* elements from the RepeatExplorer database and carrot *Alex* elements from a custom database.

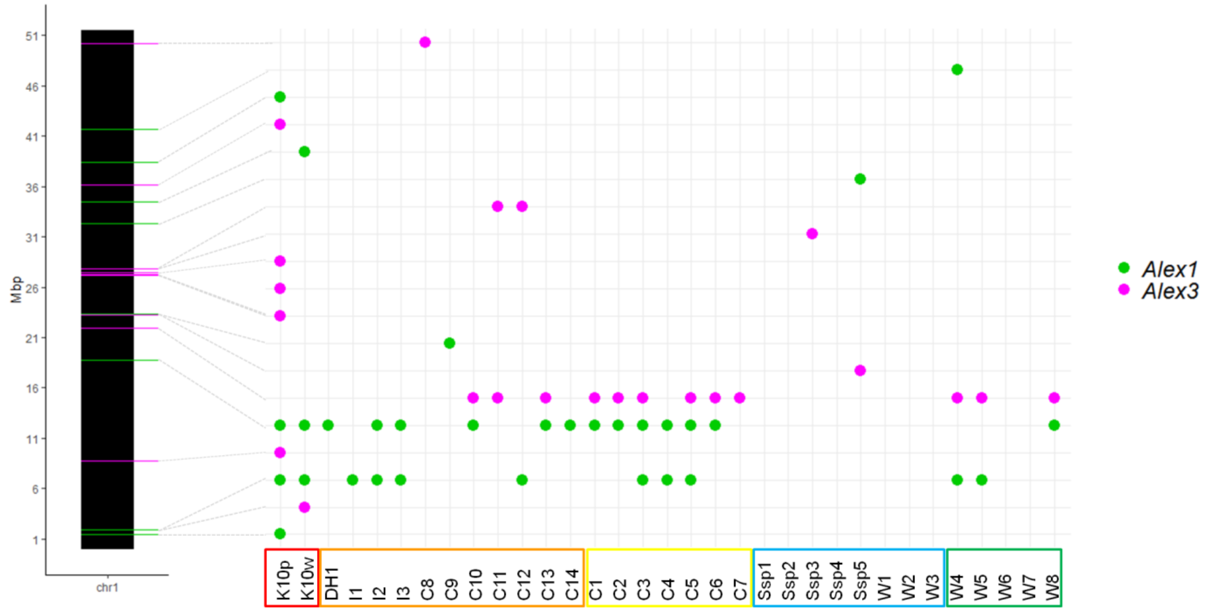

Figure S10. Localization of *Alex* copies on the carrot chromosome 1 and their distribution in cultivated and wild carrots. Groups representing K10 callus, western cultivated carrots, eastern cultivated carrots, European wild *D. carota*, and Asian wild *D. carota* are labeled with red, orange, yellow, blue, and green frames, respectively.

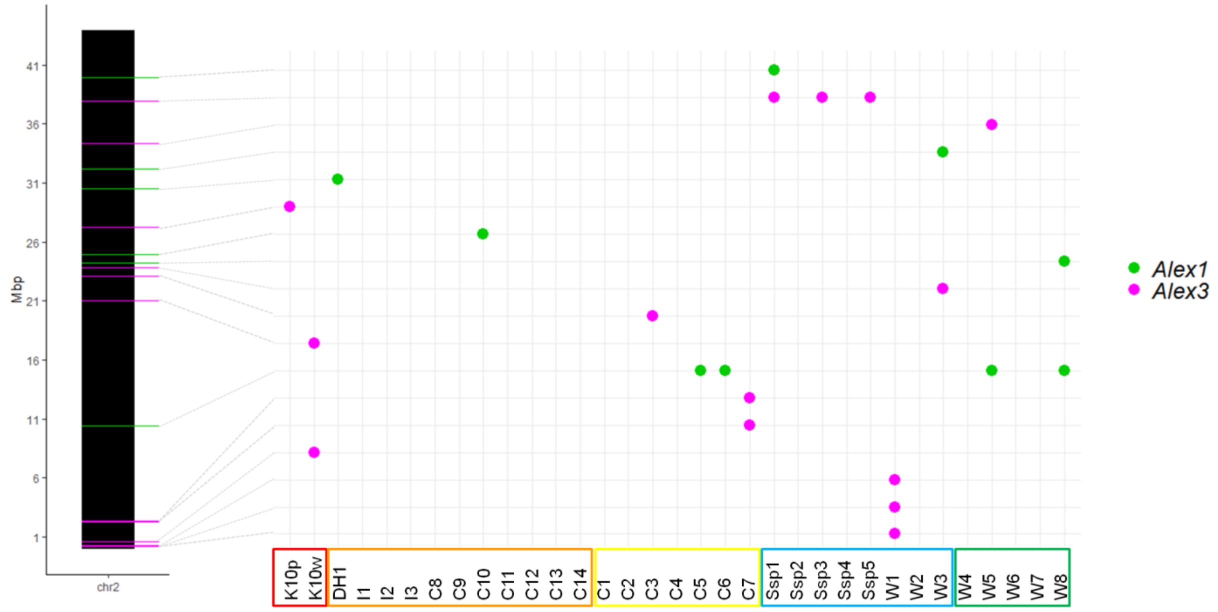

Figure S11. Localization of *Alex* copies on the carrot chromosome 2 and their distribution in cultivated and wild carrots. Groups representing K10 callus, western cultivated carrots, eastern cultivated carrots, European wild *D. carota*, and Asian wild *D. carota* are labeled with red, orange, yellow, blue, and green frames, respectively.

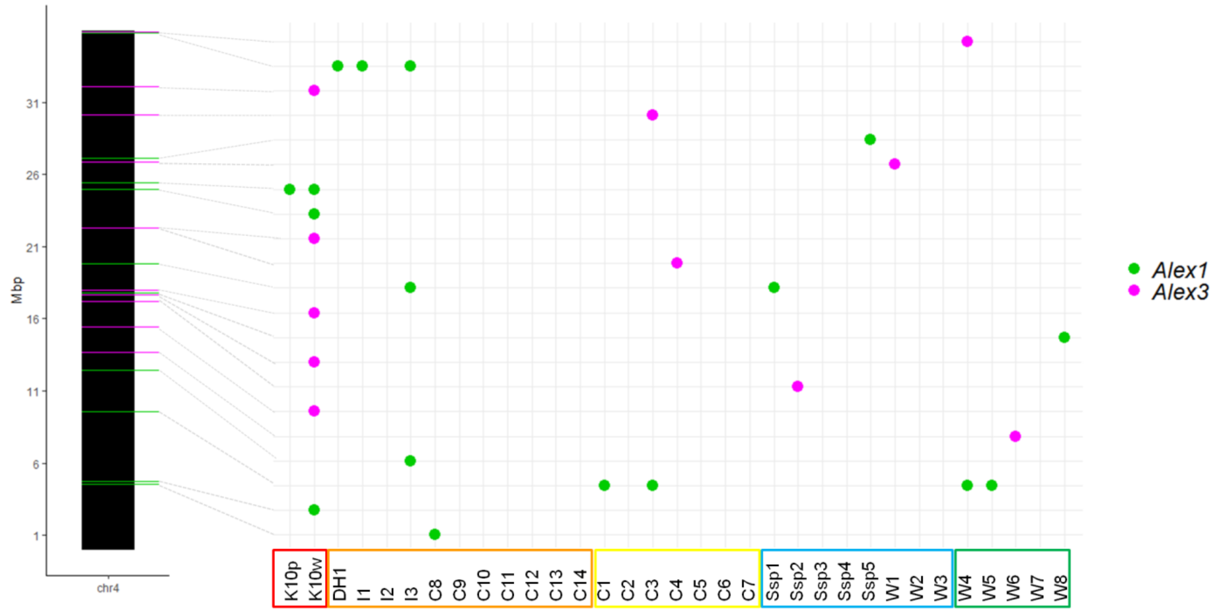

Figure S12. Localization of *Alex* copies on the carrot chromosome 4 and their distribution in cultivated and wild carrots. Groups representing K10 callus, western cultivated carrots, eastern cultivated carrots, European wild *D. carota*, and Asian wild *D. carota* are labeled with red, orange, yellow, blue, and green frames, respectively.

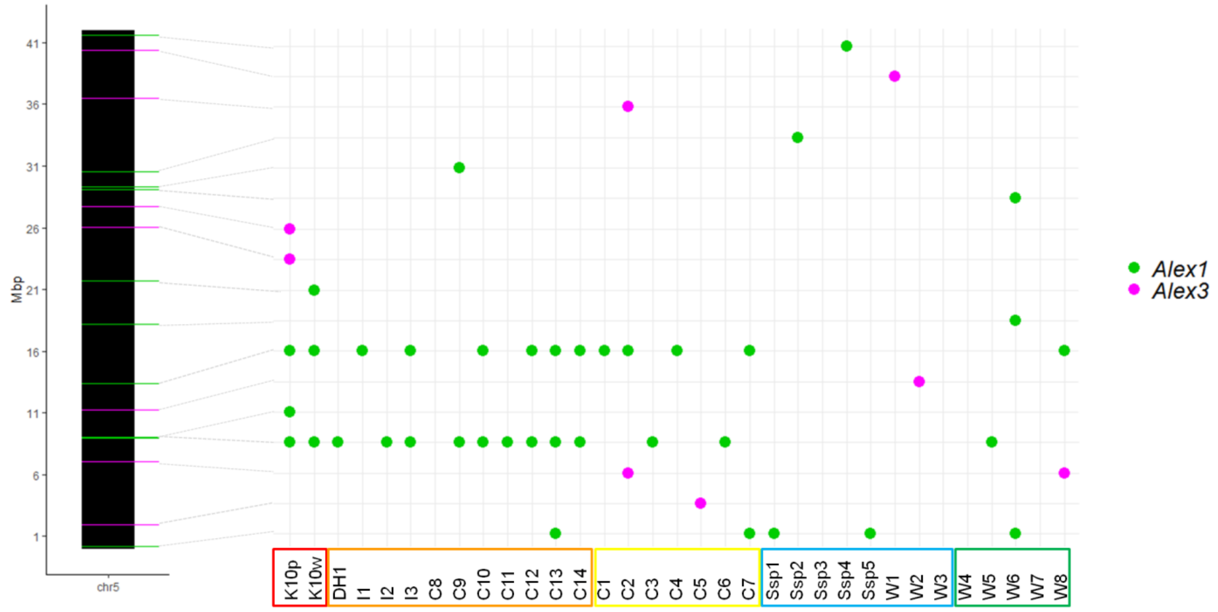

Figure S13. Localization of *Alex* copies on the carrot chromosome 5 and their distribution in cultivated and wild carrots. Groups representing K10 callus, western cultivated carrots, eastern cultivated carrots, European wild *D. carota*, and Asian wild *D. carota* are labeled with red, orange, yellow, blue, and green frames, respectively.

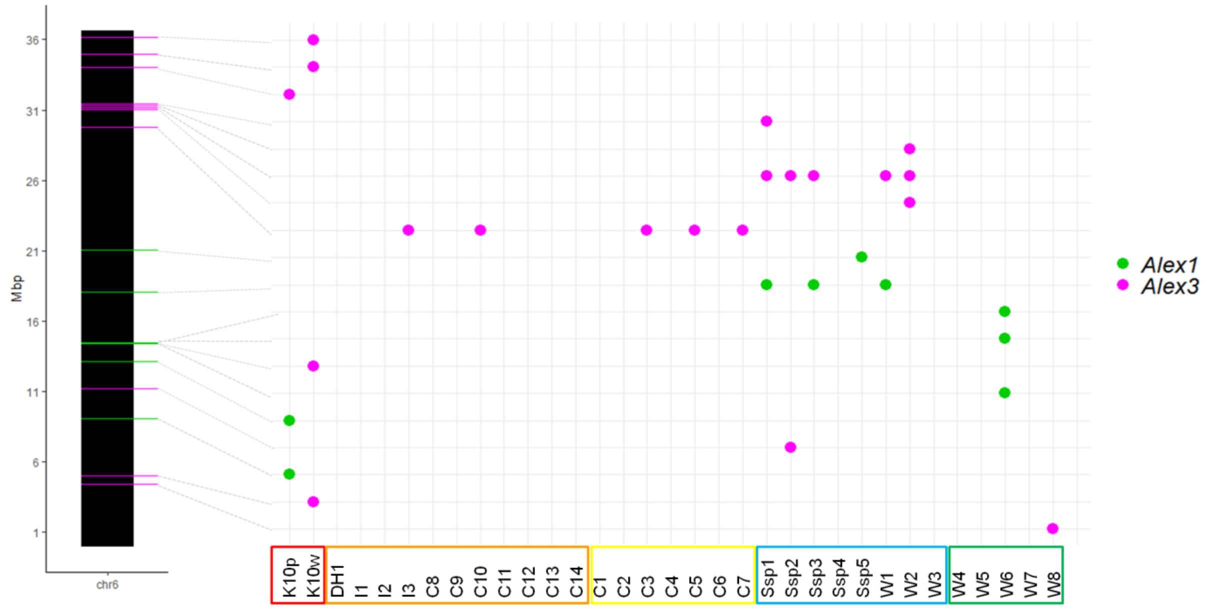

Figure S14. Localization of *Alex* copies on the carrot chromosome 6 and their distribution in cultivated and wild carrots. Groups representing K10 callus, western cultivated carrots, eastern cultivated carrots, European wild *D. carota*, and Asian wild *D. carota* are labeled with red, orange, yellow, blue, and green frames, respectively.

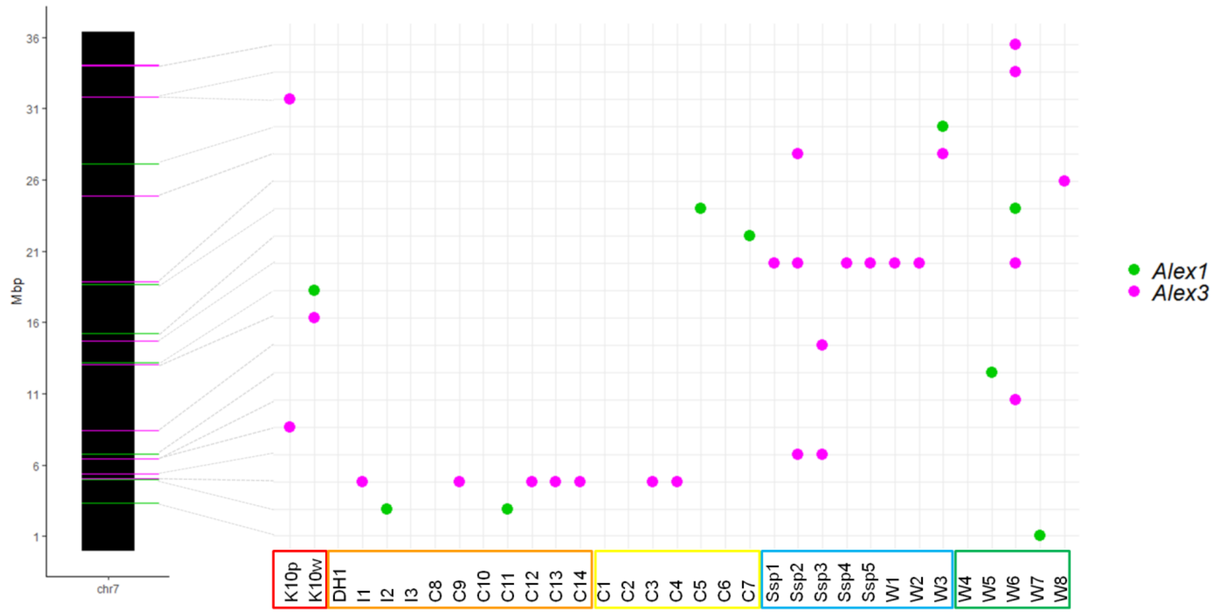

Figure S15. Localization of *Alex* copies on the carrot chromosome 7 and their distribution in cultivated and wild carrots. Groups representing K10 callus, western cultivated carrots, eastern cultivated carrots, European wild *D. carota*, and Asian wild *D. carota* are labeled with red, orange, yellow, blue, and green frames, respectively.

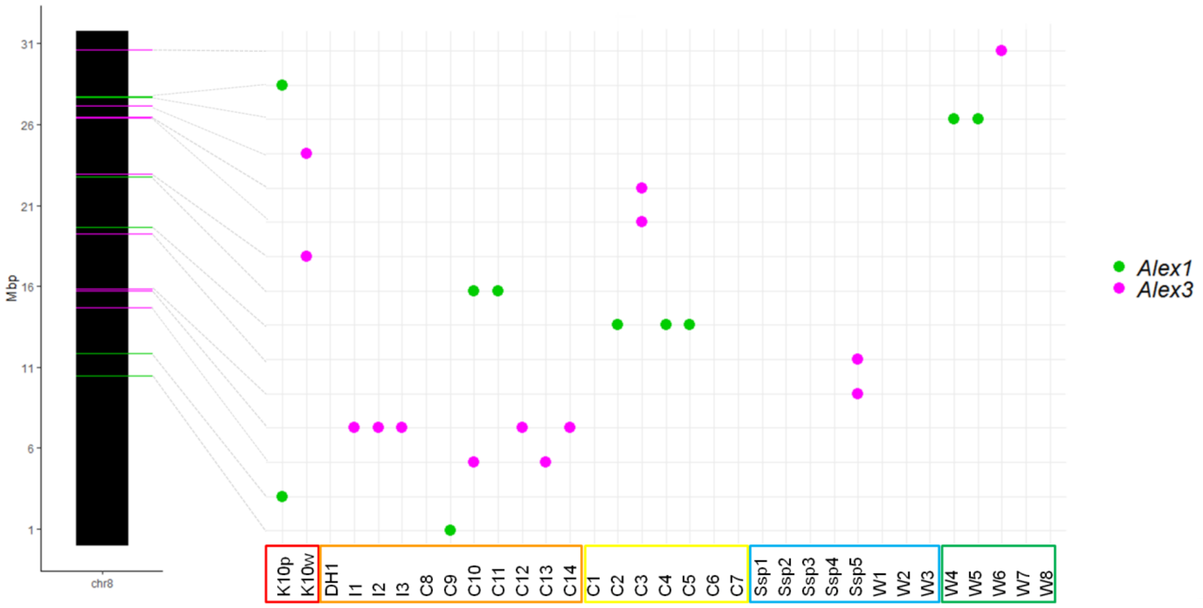

Figure S16. Localization of *Alex* copies on the carrot chromosome 8 and their distribution in cultivated and wild carrots. Groups representing K10 callus, western cultivated carrots, eastern cultivated carrots, European wild *D. carota*, and Asian wild *D. carota* are labeled with red, orange, yellow, blue, and green frames, respectively.

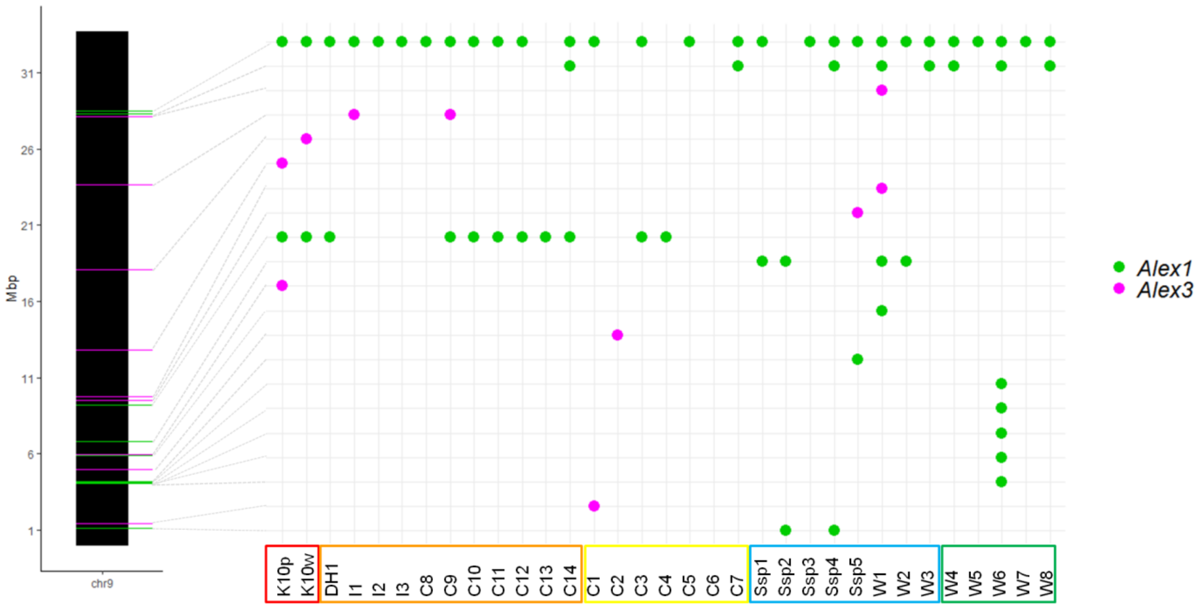

Figure S17. Localization of *Alex* copies on the carrot chromosome 9 and their distribution in cultivated and wild carrots. Groups representing K10 callus, western cultivated carrots, eastern cultivated carrots, European wild *D. carota*, and Asian wild *D. carota* are labeled with red, orange, yellow, blue, and green frames, respectively.

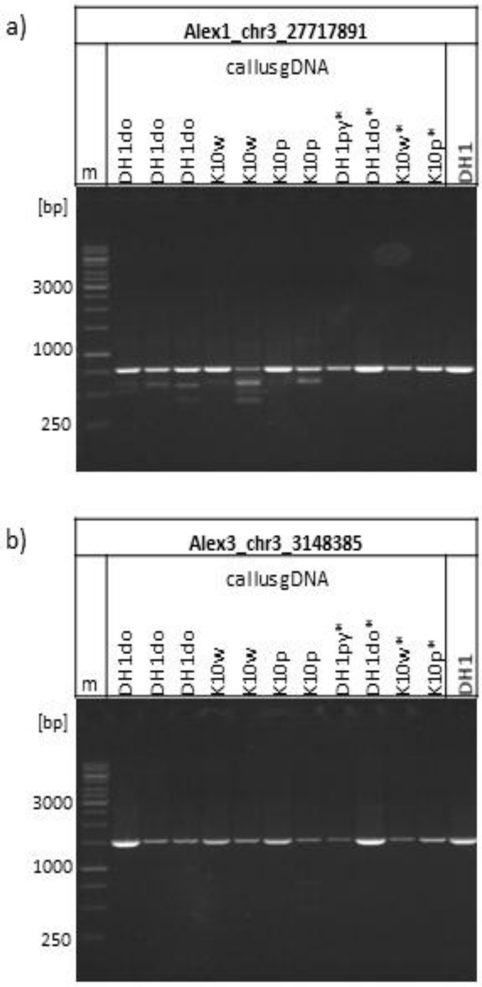

Figure S18. Reference insertions of *Alex1* and *Alex3*. Amplification of reference insertion sites of *Alex1* (a) and *Alex3* (b). Amplification from DNA samples used for eccDNA sequencing are marked with an asterisk (*). Expected size for the occupied size of *Alex1* and *Alex3* is 770 and 1536, respectively. ThermoFisher Scientific GeneRuler 1kb DNA Ladder (m) was separated along with PCR samples.

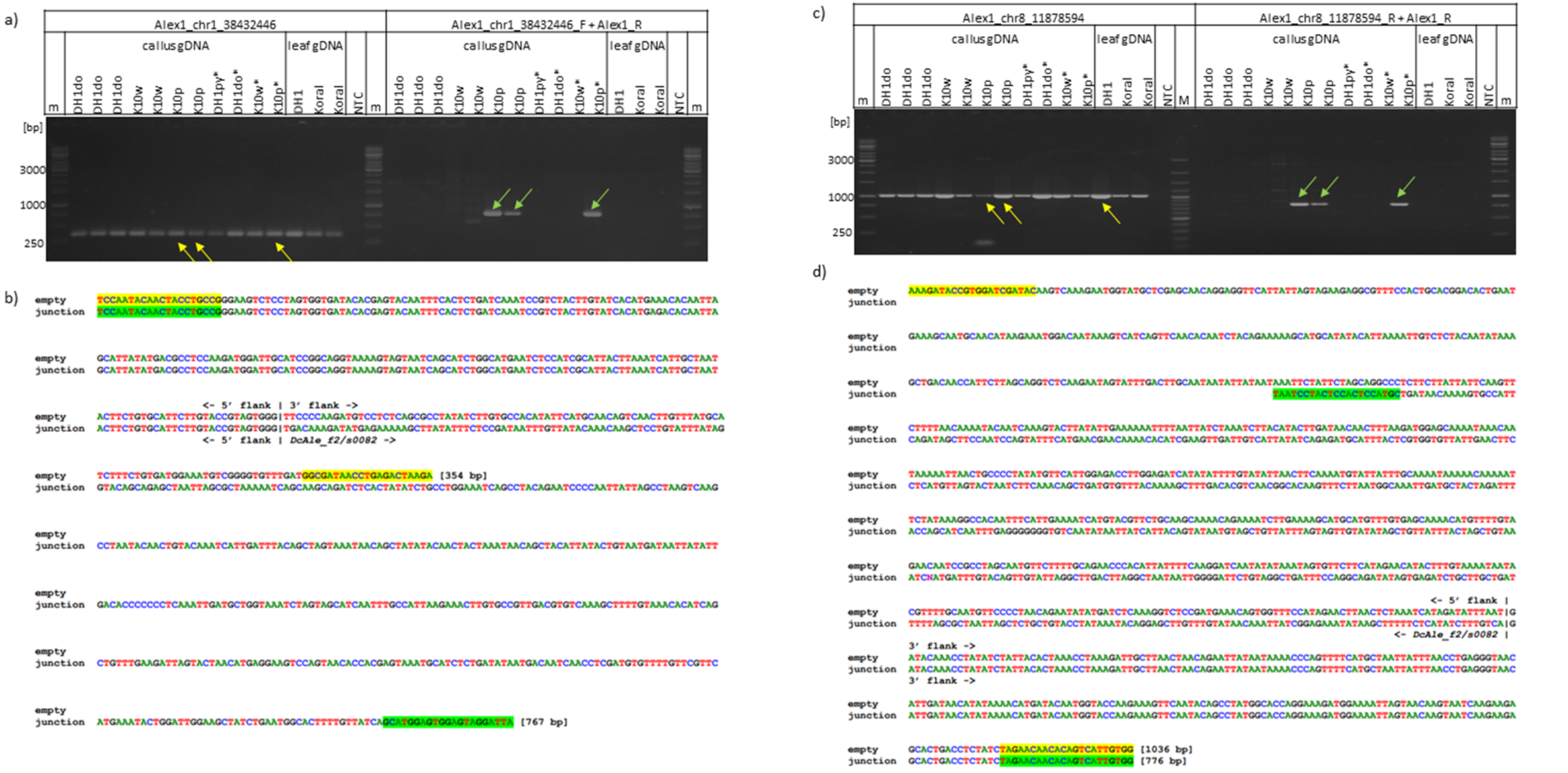

Figure S19. PCR verification of *de novo* insertion site of *Alex1* confirmed by Sanger sequencing. Results of amplification of Alex1_chr1_38432446 (a) and Alex1_chr8_11878594 (b) insertion sites and alignment of the empty site and a sequence with the fragment of *Alex1* for Alex1_chr1_38432446 (a) and Alex1_chr8_11878594 (b, d). DNA samples used for eccDNA sequencing are marked with an asterisk (*); arrows point at amplicons produced from the empty (yellow) and the occupied (green) site. The expected size of PCR amplicons is shown in parentheses, at the end of the corresponding sequence. ThermoFisher Scientific GeneRuler 1kb DNA Ladder (m) and GeneRuler 100bp Plus DNA Ladder (M) were separated along with PCR samples.

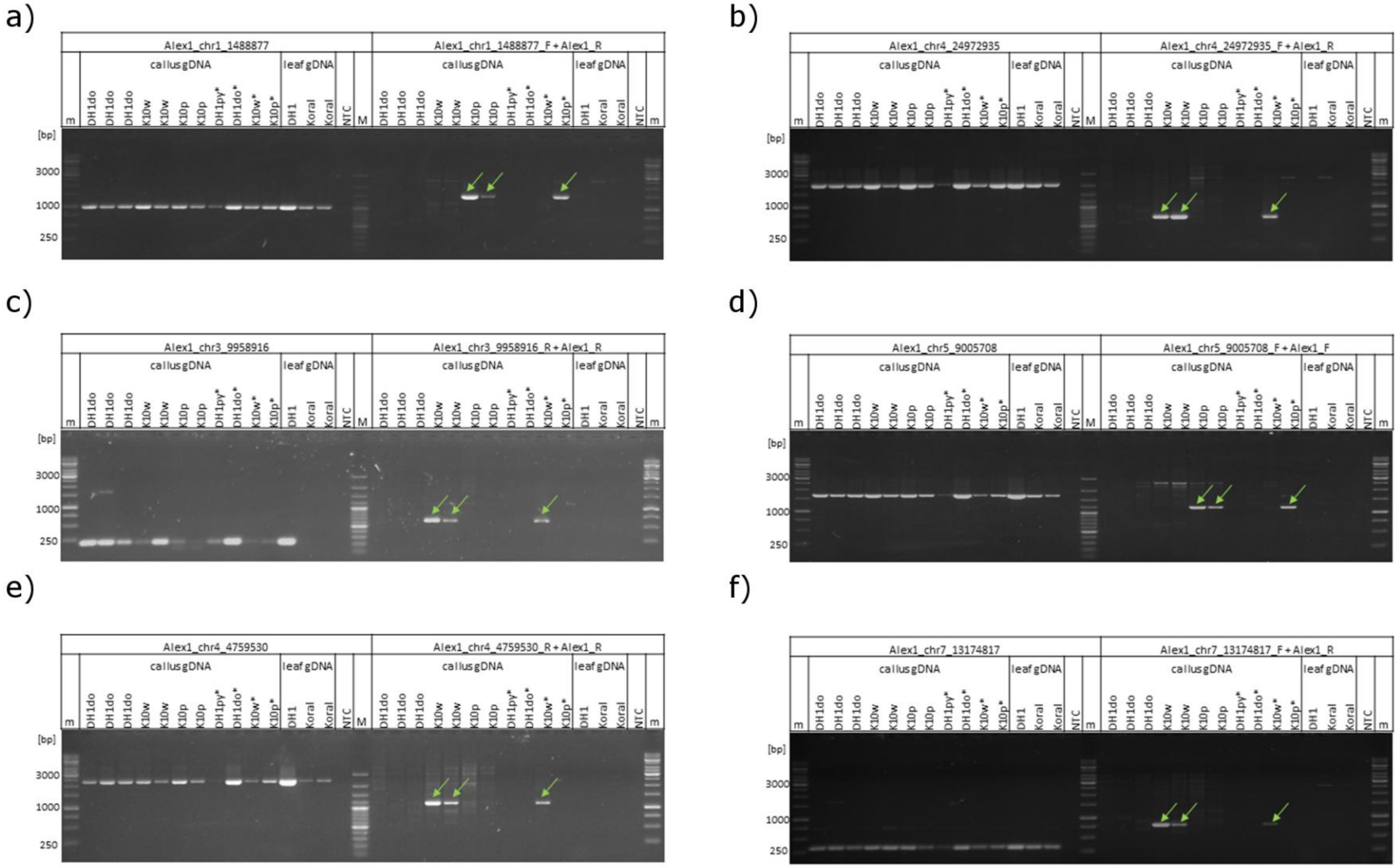

Figure S20. Amplification of *de novo* insertion sites of *Alex1*. Results of amplification of Alex1_chr1_1488877 (a), Alex1_chr4_24972935 (b), Alex1_chr3_9958916 (c), Alex1_chr5_9005708 (d), Alex1_chr4_4759530 (e), and Alex1_chr7_13174817 (f). DNA samples used for eccDNA sequencing are marked with an asterisk (*); green arrows point at amplicons produced from the occupied sites. ThermoFisher Scientific GeneRuler 1 kb DNA Ladder (m) and GeneRuler 100 bp Plus DNA Ladder (M) were separated along with PCR samples.

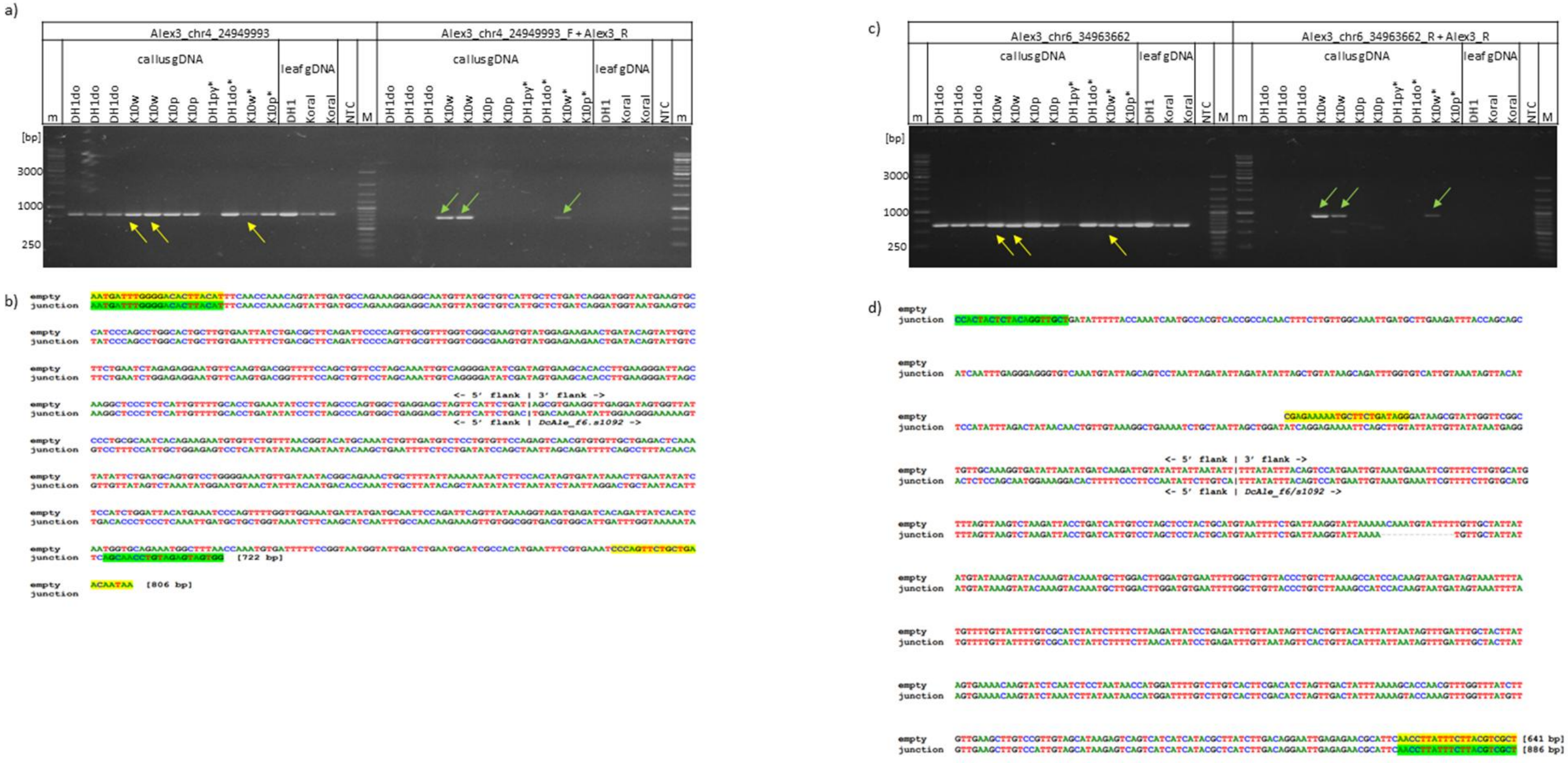

Figure S21. PCR verification of *de novo* insertion site of *Alex3* confirmed by Sanger sequencing. Results of amplification of Alex3_chr4_24949993 (a) and Alex3_chr6_34963662 (c) insertion sites and alignment of the empty site and a sequence with the fragment of *Alex3* for Alex3_chr4_24949993 (b) and Alex3_chr6_34963662 (d). DNA samples used for eccDNA sequencing are marked with an asterisk (*); arrows point at amplicons produced from the empty (yellow) and the occupied (green) site. The expected size of PCR amplicons is shown in parentheses, at the end of each sequence. ThermoFisher Scientific GeneRuler 1kb DNA Ladder (m) and GeneRuler 100bp Plus DNA Ladder (M) were separated along with PCR samples.

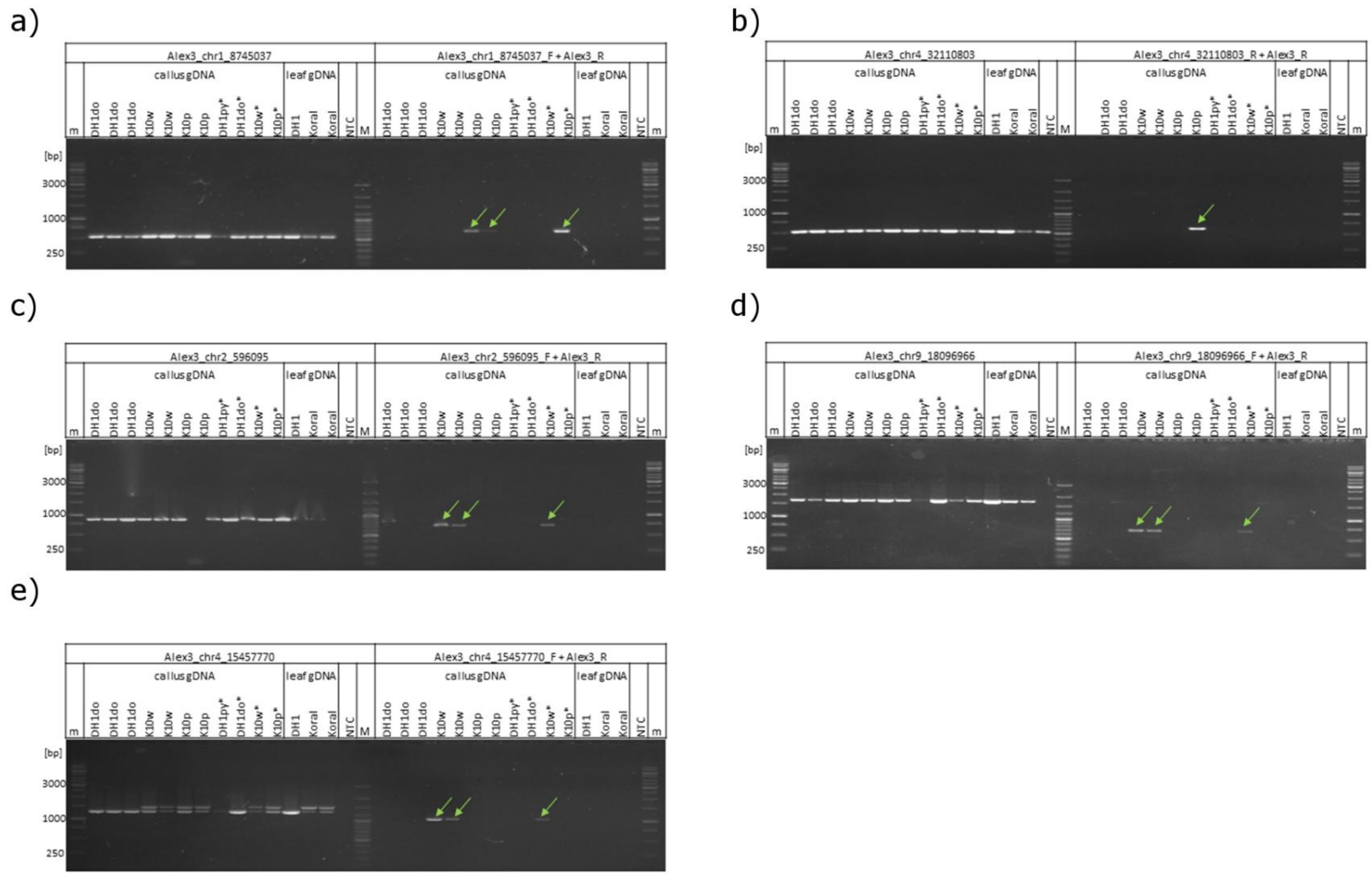

Figure S22. Amplification of *de novo* insertion sites of *Alex3*. Results of amplification of Alex3_chr1_8745037 (a), Alex3_chr4_32110803 (b), Alex3_chr2_596095 (c), Alex3_chr9_18096966 (d), and Alex3_chr4_15457770 (e). DNA samples used for eccDNA sequencing are marked with an asterisk (*); green arrows point at amplicons produced from the occupied sites. ThermoFisher Scientific GeneRuler 1 kb DNA Ladder (m) and GeneRuler 100 bp Plus DNA Ladder (M) were separated along with PCR samples.

Alex1

```
LTR - 3’end--------|    |--------LTR - 5’end
TTTTTCTCATATCTTTGTCA----ATGTCAATATAATTATCATTA (9)
TTTTTCTCATATCTTTGTCA----TTGTCAATATAATTATCATTA (6)
TTTTTCTCATATCTTTGTCA-----TGTCAATATAATTATCATTA (4)
TTTTTCTCATATCTTTGTG--------TCAATATAATTATCATTA (3)
TTTTTCTCATATCTTTGTCA---TTTGTCAATATAATTATCATTA (3)
TTTTTCTCATATCTTTGTC------TGTCAATATAATTATCATTA (2)
TTTTTCTCATATCTTTGTCA---CTTGTCAATATAATTATCATTA (2)
TTTTTCTCATATCTTTGTCA----------ATATAATTATCATTA (2)
TTTTTCTCATATCTTTGTCAA-----GTCAATATAATTATCATTA (2)
TTTTTCTCATATCTTTGTCA---ATTGTCAATATAATTATCATTA (2)
TTTTTCTCATGTCTTTGTC-------GTCAATATAATTATCATTA (1)
TTTTTCTCATATCTTCGTCAATCGCTGTCAATATAATTATCATTA (1)
TTTTTCTCATATCTTTGTCAATTTGTGTCAATATAATTATCATTA (1)
TTTTTCTCATATCTTTGTCAGTTTGTGTCAATATAATTATCATTA (1)
TTTTTCTCATATCTTTGTCGAATTGTGTCAATATAATTATCATTA (1)
TTTTTCTCATATCTTTGTCA-TTTTTGTCAATATAATTATCATTA (1)
TTTTTCTCATATCTTTGTCA---AGTGTCAATATAATTATCATTA (1)
TTTTTCTCATATCTTTGTCA--GAGTGTCAATATAATTATCATTA (1)
TTTTTCTCATATCTTTGTCA---TATGTCAATATAATTATCATTA (1)
TTTTTCTCATATCTTTGTCA--ACATGTCAATATAATTATCATTA (1)
TTTTTCTCATATCTTTGT-------TGTCAATATAATTATCATTA (1)
TTTTTCTCATATCTTTGTCA-----------TATAACTATCATTA (1)
TTTTTCTCATATCTTTGTCA-------TCAATATAATTATCATTA (1)
TTTTTC--------------------GTCAATATAATTATCATTA (1)
TTTTTCTCA---------------GTGTCAATATAATTATCATTA (1)
TTTGTC-----------------------AATATAATTATCATTA (1)
TTTTTCT------------------TGTCAATATAATTATCATTA (1)
```

Alex3

```
LTR - 3’end--------|   |--------LTR - 5’end
AACTATATATATATTTAACATATTGAAAAGAAAGCTGCTTGAA (3)
AACTATATATAT--------TATTGAAAAGAAAGCTGCTTGAA (1)
```

Figure S23. Variants of LTR-LTR junction reads identified in K10p eccDNA library. Numbers of reads corresponding to each variant are given in parentheses.

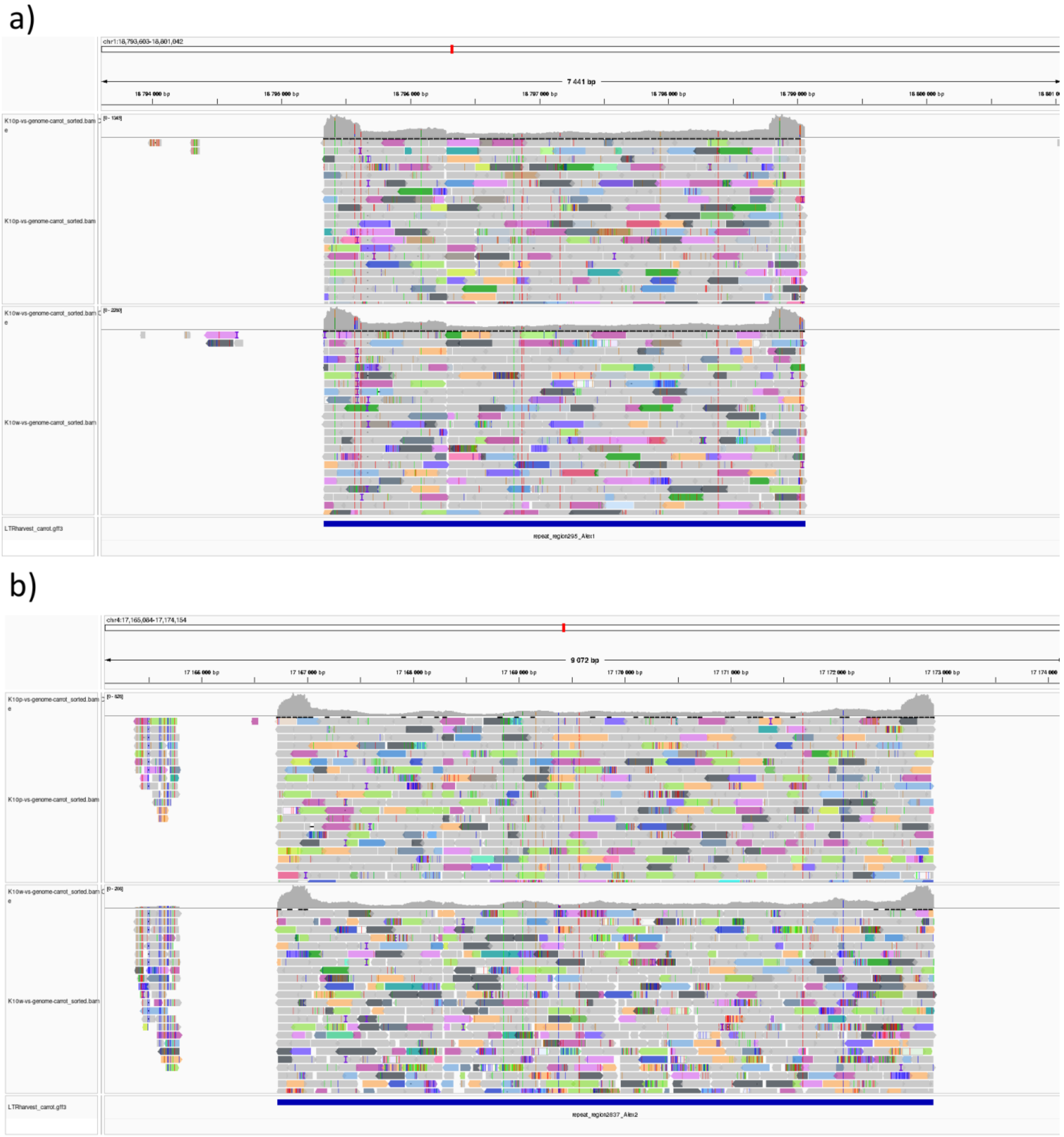

Figure S24. IGV view of alignments of eccDNA reads derived from K10w and K10p callus sub-lines to copies of *Alex1* (a) and *Alex3* (b) in the carrot reference genome DH1.

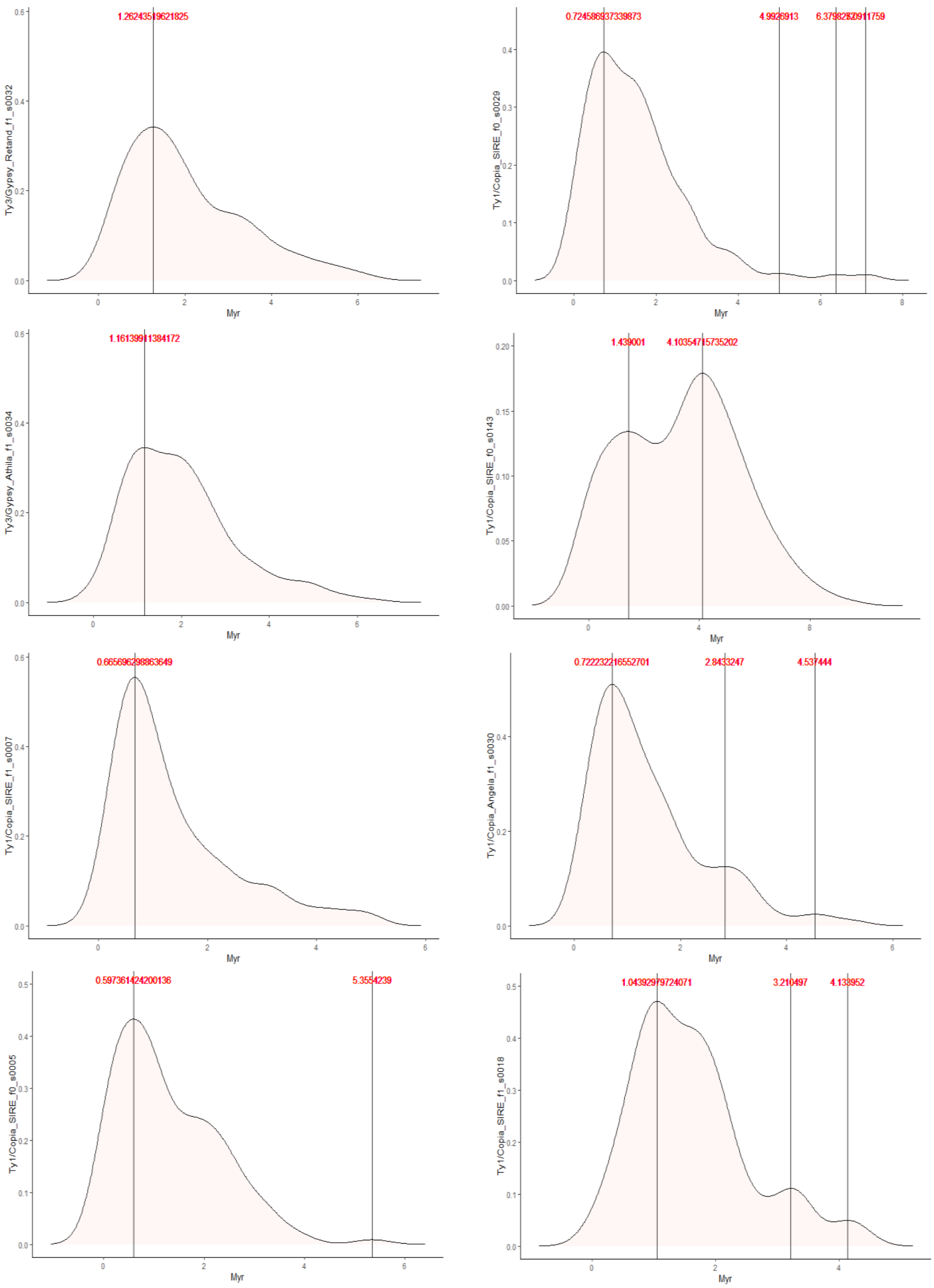

Figure S25. Density plots showing the age distribution of carrot LTR-RTs representing eight most numerous subfamilies (containing more than 100 copies). Modes are indicated by vertical lines.
