# Supplementary material for: Diverse and mobile: eccDNA‐based identification of carrot low‐copy‐number LTR retrotransposons active in callus cultures
Source: Plant J. 2022 May 10;110(6):1811–28. doi: 10.1111/tpj.15773 (PMC9324142; doi:10.1111/tpj.15773)
Supplement: Supplementary file 2 — Figure S3. Sunburst chart for low‐ (≤10) and high (>10) copy number subfamilies (inner ring), their localization in the genome (middle ring), and their classification into families (outer ring). More details are provided in the html version of the figure (Figure_S3.html). [file TPJ-110-1811-s002.html]

Legend
