# Supplementary material for: Diverse and mobile: eccDNA‐based identification of carrot low‐copy‐number LTR retrotransposons active in callus cultures
Source: Plant J. 2022 May 10;110(6):1811–28. doi: 10.1111/tpj.15773 (PMC9324142; doi:10.1111/tpj.15773)
Supplement: Supplementary file 3 — Table S1. Superfamilies of LTR‐RTs identified in the carrot DH1 reference genome. Table S2. Characteristics of LTR‐RT lineages in the carrot DH1 reference genome. Table S3. Mapping statistics of eccDNA reads to the carrot DH1 reference genome. Table S4. Statistics of eccDNA read assemblies. Table S5. Summary of repeatexplorer clusters annotation. Table S6. Characteristics of LTR‐RTs overrepresented in mobilomes of four carrot callus sublines lines identified based on the repeatexplorer comparative analysis. Table S7. Number and proportion of reads attributed to each carrot LTR‐RT after the merging of clusters representing individual elements. Table S8. Characterization of LTR‐RT superfamilies identified in eccDNA, including clusters representing each superfamily, domains detected by repeatexplorer , age and abundance of superfamily based on copies in the reference genome. Table S9. Summary statistics of callus subline sample sequencing results. Table S10. Validation of the performance of the modified trackposon pipeline. Table S11. Summary of de novo insertion sites identified for LTR‐RT enriched in eccDNA. Table S12. Insertion sites of Alex1 in K10p and K10w callus sublines. Table S13. Insertion sites of Alex2 in K10w and K10p callus sublines. Table S14. Insertion sites of Alex3 in K10w and K10p callus sublines. Table S15. Insertion sites of Ivan1 in K10w and K10p callus sublines. Table S16. Insertion sites of DcTork_f0/s1917 in K10w and K10p callus lines. Table S17. Insertion sites of DcTork_f1/s2099 in K10w and K10p callus sublines. Table S18. List of Daucus carota accessions with resequenced genomes. Table S19. Summary of the verification of de novo insertion sites of Alex1 in K10p and K10w callus sublines. Table S20. Summary of the verification of de novo insertion sites of Alex3 in K10w and K10p callus sublines. Table S21. Primers for verification of LTR‐RT circularization. Table S22. Primers for verification of the presence of the LTR‐RTs domain transcripts a [file TPJ-110-1811-s003.pdf]

Table S1. Superfamilies of LTR-RTs identified in the carrot DH1 reference genome

Classification	Number of families	Number of subfamilies	Number of copies	Copies on assembled chromosomes
**Order: LTR**	**86**	**1140**	**3713**	**2889 (78%)**
Ty1/copia	71	588	2053	1587 (77%)
Ty3/gypsy|non-chromovirus	8	242	1138	837 (77%)
Ty3/gypsy|chromovirus	7	310	522	469 (82%)
**Order: LTR - unclassified**	**-**	**-**	**2263**	**1849 (82%)**
**Total**	**-**	**-**	**5976**	**4738 (80%)**

Table S2. Characteristics of LTR-RT lineages in carrot DH1 reference genome

Family	Total number of intact copies	Number of single copies	Total number of soloLTRs	soloLTRs / intact copies	Number of families†	Number of subfamilies	Mean of age (Myr) and confidence intervals (p=0.05)‡	Number copies of the most numerous subfamily	Intact LTR-RTs				soloLTRs			
									Intergenic	Upstream	Downstream	Genic	Intergenic	Upstream	Downstream	Genic
Ty1/Copia:																
DcAle	241	124	90	0.37	38	174	1.14 (0.95-1.32)	8	169	15	15	42	62	15	15	17
DcAlesia	8	5	2	0.25	4	6	1.44	3	7	0	0	1	0	0	0	2
DcAngela	115	14	556	4.83	3	17	1.38 (1.25-1.52)	73	108	1	1	5	523	1	1	17
DcBianca	91	14	81	0.89	2	22	0.82 (0.60-1.04)	29	60	9	3	10	65	9	3	6
DcIkeros	7	3	5	0.71	2	5	1.26	2	6	0	0	1	4	0	0	1
DcIvana	154	100	94	0.61	20	121	1.20 (1.01-1.40)	5	127	5	8	14	71	5	8	20
DcSIRE	897	85	5701	6.36	2	113	1.70 (1.62-1.78)	185	806	26	23	42	5289	26	23	239
DcTAR	104	18	206	1.98	6	29	1.21 (0.96-1.45)	19	76	9	5	14	150	9	5	31
DcTork	41	15	45	1.1	5	19	0.88 (0.60-1.17)	18	27	4	3	7	29	4	3	8
Ty3/gypsy|chromovirus:																
DcCRM	16	6	23	1.44	2	10	0.84 (0.39-1.31)	4	11	1	0	4	14	1	0	6
DcGaladriel	2	2	0	-	2	2	1.15	1	2	0	0	0	0	0	0	0
DcReina	146	76	18	0.12	2	104	0.64 (0.49-0.80)	8	86	9	12	39	8	9	12	10
DcTekay	265	126	1011	3.82	1	152	1.57 (1.42-1.72)	29	227	2	3	33	905	2	3	67
Ty3/gypsy|non-chromovirus:																
DcAthila	309	58	1411	4.57	6	79	1.61 (1.50-1.72)	139	269	4	9	27	1218	4	9	120
DcRetand	493	88	1054	2.14	2	121	1.24 (1.14-1.33)	185	427	22	20	24	884	22	20	73
**LTR classified**	**2889**	**-**	**10297**	**-**	**-**	**-**	-	**-**	**2417**	**107**	**102**	**263**	**9222**	**107**	**102**	**617**
**LTR unclassified**	**1849**	**-**	**2810**	**-**	**-**	**-**	**2.16 (2.08-2.24)**	**-**	**1349**	**42**	**18**	**440**	**2073**	**42**	**18**	**308**

† including f0, grouping all subfamilies not meeting the criteria for phylogenetic analysis

‡ confidence intervals are shown for lineages represented by more than 10 copies

Table S3. Mapping statistics of eccDNA reads to the carrot DH1 reference genome

Sample name	Number of paired reads	Number of reads after filtering	Alignment to the genome [%]	Alignment to LTR-RTs [%]
DH1py	510885	509286	70.75	9.82
DH1do	542411	540924	67.01	11.46
K10w	651930	650149	69.71	20.27
K10p	482978	482475	67.95	23.20

Table S4. Statistics of eccDNA read assemblies

Sample name	Number of raw reads	Longest contig [bp]	N50 [bp]	Number of contigs	Contigs mapping to LTR-RTs [meeting the 90/80 criterion]
DH1py	1021770	15720	1418	566	115
DH1do	1084822	22807	1789	713	124
K10w	1303860	12884	1594	856	137
K10p	965956	16987	2004	554	96

Table S5. Summary of RepeatExplorer clusters annotation

Annotation	No. clusters	Proportion [%]	No. reads
Ale	12	4.25	21234
Angela	1	0.03	130
Bianca	2	0.29	1430
Ivana	4	0.36	1781
SIRE	6	0.18	908
TAR	7	2.00	9977
Tork	5	0.88	4418
Reina	4	0.43	2146
Organelle (plastid, mtochondia)	150	22.56	112784
rDNA	5	0.28	1381
Unclassified repeats	65	0.19	30946

Table S6. Characteristics of LTR-RTs overrepresented in mobilomes of two pairs of carrot callus sub-lines identified based on the RepeatExplorer comparative analysis

Cluster	Cluster size	Automatic_annotation	Fam/Subfam	RE counts K10w	RE counts K10p	Koral p.adj. Chisq	RE counts DH1do	RE counts DH1py	DH1 p.adj.Chisq	4 lines p.adj.Chisq
5	3394	Class_I/LTR/Ty1_copia/Ale	DcAle_f2/s0082 (DcAle_1.7)	1342	814	2.94e-06	922	316	1.52e-56	3.98e-88
47	1548	Class_I/LTR/Ty1_copia/Ale	DcAle_f2/s0082 (DcAle_1.7)	696	517	0.9	139	196	0.0013	3.56e-94
35	1887	Class_I/LTR/Ty1_copia/Ale	DcAle_f0/s0191 (DcAle_28.1)	501	1324	1.13e-142	48	14	0.000262	0
76	929	Class_I/LTR/Ty1_copia/Ale	DcAle_f1/s0318 (DcAle_36.2)	192	652	2.25e-89	31	54	0.0202	9.59e-275
15	2425	Class_I/LTR/Ty1_copia/Ale	DcAle_f6/s1092 (DcAle_84)	863	1467	5.38e-84	66	29	0.00196	0
44	1636	Class_I/LTR/Ty1_copia/Ale	DcAle_f1/s0318 (DcAle_84)	545	985	9.94e-64	88	18	1.19e-09	0
65	1141	Class_I/LTR/Ty1_copia/Ale	DcAle_f1/ s0318 (DcAle_84)	345	734	1.36e-61	40	22	0.1	6.58e-288
87	756	Class_I/LTR/Ty1_copia/Bianca	DcBianca_f1/s1628 (DcBianca_19)	100	16	7.46e-10	495	145	3.62e-37	3.12e-155
96	674	Class_I/LTR/Ty1_copia/Bianca	DcBianca_f1/s1628 (DcBianca_19)	105	14	2.3e-11	388	167	5.24e-17	1.04e-99
51	1453	Class_I/LTR/Ty1_copia/Ivana	DcIvana_f6/s0395 (DcIvana_24.2)	1266	17	1.83e-196	163	7	3.88e-29	0
13	2613	Class_I/LTR/Ty1_copia/TAR	DcTAR_f0/s1350 (DcTAR_18)	1855	422	7.73e-120			8.26e-39	0
55	1384	Class_I/LTR/Ty1_copia/TAR	DcTAR_f0/s1350 (DcTAR_18)	1004	217	3.99e-69	147	16	1.5e-21	1.37e-276
64	1172	Class_I/LTR/Ty1_copia/TAR	DcTAR_f0/s1350 (DcTAR_18)	845	195	1.06e-54	115	17	5.6e-15	8.66e-231
21	2191	Class_I/LTR/Ty1_copia/TAR	DcTAR_f0/s2199 (DcTAR_27)	1600	346	4.34e-109	217	28	2.53e-29	0
30	2002	Class_I/LTR/Ty1_copia/TAR	DcTAR_f0/s2199 (DcTAR_27)	1455	323	8.12e-97	198	26	8.00e-27	0
50	1476	Class_I/LTR/Ty1_copia/Tork	DcTork_f0/s1917 (DcTork_16)	25	1433	0	8	10	0.854	0

74	965	Class_I/LTR/Ty1_copia/Tork	DcTork_f1/s2099 (DcTork_17)	221	714	7.21e-94		0.0225	0

Table S7. **Number and proportion of reads attributed to each carrot LTR-RT after merging of clusters representing individual element**

subfamily	clusters	RE counts K10w	RE counts K10p	RE counts DH1do	RE counts DH1py
DcAle_f2/s0082 (Alex1)	5,47	2038 (41%)	1331 (27%)	1061 (21%)	512 (10%)
DcAle_f1/s0318 (Alex2)	76	192 (21%)	652 (70%)	31 (3%)	54 (6%)
DcAle_f6/s1092 (*Alex3*)	15,44,65	1753 (34%)	3186 (61%)	194 (4%)	69 (1%)
DcAle_f0/s0191	76	501 (27%)	1324 (70%)	48 (7%)	14 (1%)
DcIvana_f6/s0395 (*Ivan1*)	51	1266 (87%)	17 (1%)	163 (11%)	7 (1%)
DcBianca_f1/s1628	87,96	205 (14%)	30 (2%)	883 (62%)	312 (22%)
DcTAR_f0/s1350	13,55,64	3704 (78%)	834 (18%)	187 (4%)	33 (1%)
DcTAR_f0/s219	21,30	3055 (73%)	669 (16%)	415 (10%)	54 (1%)
DcTork_f0/s1917	50	25 (2%)	1433 (97%)	8 (1%)	10 (1%)
DcTork_f1/s2099	74	221 (22%)	714 (70%)	74 (7%)	7 (1%)

Table S8. Characterisation of LTR-RT superfamilies identified in eccDNA including clusters representing each superfamily, domains detected by RepeatExplorer, age and abundance of superfamily based on copies in the reference genome.

Subfamily	Cluster	GAG	PROT	INT	RT	RH	Superfamily age (Myr) based on copies in reference genome	Copies in reference genome
DcAle_f2/s0082 (Alex1)	5,47	+	+	+	+	+	0.39 (0-1.39)	8
DcAle_f1/s0318 (Alex2)	76	+	+	+	+	+	0.38 (0-0.77)	2
DcAle_f6/s1092 (Alex3)	15,44,65	+	+	+	+	+	0	1
DcAle_f0/s0191	76	+	+	+	+	+	0.62 (0.26-0.98)	2
DcIvana_f6/s0395 (Ivan1)	51	+	+	+	+	+	0.90 (1.48-0.31)	2
DcBianca_f1/s1628	87,96	+	+	+	+	+	0	1
DcTAR_f0/s1350	13,55,64	+	+	+	-	+	0	1
DcTAR_f0/s219	21,30	-	-	+	+	+	0.76 (0.36-1.74)	8
DcTork_f0/s1917	50	+	+	+	+	+	0.96	1
DcTork_f1/s2099	74	+	+	+	+	-	0.11	1

Table S9. Summary statistics of callus samples sequencing results

Sample	Mode	Mapped reads	Total reads	Coverage
DH1do	PE (150)	99.65%	90308819	~28x
K10w	PE (150)	98.35%	91524906	~29x
K10p	PE (150)	98.38%	59607033	~19x

Table S10. Validation of the performance of the modified TRACKPOSON pipeline. Calls representing false-positive and false-negative are highlighted in red and yellow, respectively

Alex	Chr.	Beginning of bin	Localization	TRACKPOSON				TRACKPOSON (modified, unmasked genome)				TRACKPOSON (modified, masked genome)			
				DH1	DH1do	K10w	K10p	DH1	DH1do	K10w	K10p	DH1	DH1do	K10w	K10p
Alex1	1	18790000	ref:(masked) intron	+	+	+	+	-	-	-	-	+	+	+	+
Alex1	2	30530000	ref: intergenic	+	+	-	-	-	-	-	-	-	-	-	-
Alex1	3	27710000	ref(masked): intron	+	+	+	+	-	-	-	-	+	+	+	+
Alex1	3	41150000	ref(masked): intron	+	+	+	+	-	-	-	-	-	+	+	+
Alex1	4	35820000	ref: intron	+	+	-	-	-	-	-	-	-	-	-	-
Alex1	5	8960000	ref(masked): intron	+	+	+	+	-	-	-	-	+	+	+	+
Alex1	9	9220000	ref(masked): intron	+	+	+	+	-	-	+	-	+	+	+	+
Alex1	9	28520000	ref(masked): intron	+	+	+	+	-	-	-	-	+	+	+	+
Alex3	3	3150000	ref(masked): intron/downstream	+	+	+	+	-	-	+	-	+	+	+	+

Table S11. **Summary of *de novo* insertion sites identified for LTR-RT enriched in eccDNA**

LTR-RT	RepeatExplorer clusters	DH1	DH1do	K10w	K10p
DcAle_f2/s0082 (Alex1)	5,47	0	0	**6**	**8**
DcAle_f1/s0318 (Alex2)	76	0	0	**5**	0
DcAle_f6/s1092 (Alex3)	15,44,65	0	0	**19**	**13**
DcAle_f0/s0191	76	0	0	0	0
DcIvana_f6/s0395 (Ivan1)	51	0	0	**19**	0
DcBianca_f1/s1628	87,96	0	0	0	0
DcTAR_f0/s1350	13,55,64	0	0	0	0
DcTAR_f0/s219	21,30	0	0	0	0
DcTork_f0/s1917	50	0	0	0	**2**
DcTork_f1/s2099	74	0	0	0	**7**

Table S12. Insertion sites of *Alex1* in K10p and K10w callus sub-lines. DH1 reference insertion sites of intact copies identified in K10 callus are bolded

Chr.	Beginning of bin	Localization	Gene ID	Gene function	Callus sub-line
chr1	1480000	exon	LOC108200820	nuclear mitotic apparatus protein 1	K10p
chr1	1890000	intron	LOC108193497	uncharacterized LOC108193497	K10w,K10p
**chr1**	**18790000**	**ref:(masked) intron**	**LOC108204518**	**triacylglycerol lipase 1**	**K10w, K10p**
chr1	34500000	intron	LOC108204229	alpha-mannosidase	K10w
chr1	38430000	exon	LOC108204091	topless-related protein 3-like	K10p
chr3	9950000	intron	LOC108214234	importin-5-like	K10w
**chr3**	14350000	intron	LOC108215009	tRNA threonylcarbamoyladenosine dehydratase	K10p
**chr3**	**27710000**	**ref(masked): intron**	**LOC108214167**	**protease Do-like 7**	**K10w, K10p**
chr3	**41150000**	**ref(masked): intron**	**LOC108214132**	**cytosolic endo-beta-N-acetylglucosaminidase 1**	**K10w, K10p**
chr4	4750000	5’ UTR	LOC108215752	putative F-box protein At3g23950	K10w
chr4	24970000	exon	LOC108219529	small glutamine-rich tetratricopeptide repeat-containing protein	K10w
chr4	25430000	5' UTR	LOC108219015	protein LONGIFOLIA 1-like	K10w, K10p
**Chr5**	**8960000**	**ref(masked): intron**	**LOC108222702**	**callose synthase 7-like**	**K10w, K10p**
chr5	9000000	intron	LOC108223089	ubiquitin carboxyl-terminal hydrolase 12-like	K10p
chr5	13410000	intron	LOC108220574	HVA22-like protein k	K10w, K10p
chr5	21720000	intron	LOC108223602	pyrophosphate--fructose 6-phosphate 1-phosphotransferase subunit alpha	K10w
chr6	9100000	intron	LOC108224334	probable complex I intermediate-associated protein 30	K10p
chr6	13140000	intron	LOC108225164	DNA-directed RNA polymerases II, IV and V subunit 11	K10p
chr7	13170000	exon	LOC108195910	callose synthase 3-like	K10w
chr8	11870000	Intron	LOC108199531	tip elongation aberrant protein 1	K10p
chr8	27700000	intron	LOC108199983	MAP3K epsilon protein kinase 1	K10p
**chr9**	**9220000**	**ref(masked): intron**	**LOC108202424**	**mediator of RNA polymerase II transcription subunit 13**	**K10w, K10p**
**chr9**	**28520000**	**ref(masked): intron**	**LOC108202758**	**uncharacterized**	**K10w, K10p**

Table S13. Insertion sites of *Alex2* in K10p and K10w callus sub-lines. DH1 reference insertion sites of intact copies identified in K10 callus are bolded

Chr.	Beginning of bin	Localization	Gene ID	Gene function	Callus sub-line
**chr4**	**17160000**	**ref: intron/exon**	**LOC108217263**	**uncharacterized**	**K10w,K10p**
chr4	23360000	intergenic	-	-	K10w
chr5	320000	intergenic/TE	-	-	K10w
chr5	13550000	intergenic/TE	-	-	K10w
chr5	25610000	intergenic/TE	-	-	K10w, K10p
**chr6**	**17450000**	**ref: intergenic**	**-**	**-**	**K10w,K10p**
chr9	31300000	intergenic/TE	-	-	K10w
chr9	31310000	intergenic/TE	-	-	K10w

Table S14. Insertion sites of *Alex3* in K10p and K10w callus sub-lines. DH1 reference insertion sites of intact copies identified in K10 callus are bolded

Chr.	Beginning of bin	Localization	Gene ID	Gene function	Callus sub-line
chr1	1890000	intron	LOC108193497	uncharacterized	K10w
chr1	8740000	intron	LOC108204794	phragmoplast orienting kinesin 2	K10p
chr1	27190000	5'UTR/TE annot	LOC108193248		K10p
chr1	27200000	intron/TE annot	LOC108193289		K10p
chr1	27240000	intergenic/TE annot	-	-	K10p
chr1	36150000	intergenic	-	-	K10p
chr2	590000	intron	LOC108209006	ABC transporter C family member 12-like	K10w
chr2	21010000	3'UTR/Exon/Intron	LOC108205636	L-ascorbate peroxidase, cytosolic-like	K10w
chr2	27260000	intron	LOC108208894	uncharacterized	K10p
**chr3**	**3150000**	**ref: intron/downstream**	**LOC108214759/DCAR_008967**	**uncharacterized**	**K10w, K10p**
chr3	32550000	intron	LOC108214249	pleiotropic drug resistance protein 3-like	K10w
chr3	32560000	5'UTRLOC/intron DCAR/gaps	LOC108214246	pleiotropic drug resistance protein 3-like	K10w
chr3	32590000	intron	LOC108214248	pleiotropic drug resistance protein 3-like	K10w
chr4	15450000	intron	LOC108217558	probable acyl-activating enzyme 17, peroxisomal	K10w
chr4	17680000	intron/exon splice-variants	LOC108215775	lncRNA	K10w
chr4	17990000	intergenic	-	-	K10w
chr4	24940000	exon	LOC108215858	uncharacterized	K10w
chr4	32110000	intron (plant Tfcat?)	LOC108218452	histone acetyltransferase HAC1-like	K10w
chr5	26040000	3'UTR/exon (Tfcat_WRKY)	LOC108220132	probable WRKY transcription factor 70	K10p
chr5	27750000	intron/exon (ong, rep)	LOC108223306	paladin-like	K10p
chr6	4990000	3'UTR	LOC108225424	pre-mRNA-splicing factor CWC25 homolog	K10w

chr6	14420000	TE/gap/intergenic	-	-	K10w
chr6	34050000	intron	LOC108227384	FGGY carbohydrate kinase domain-containing protein	K10p
chr6	34960000	intron/Tfcat_SNF2	LOC108227973	protein CHROMATIN REMODELING 24	K10w
chr6	36180000	3'UTR/exon	LOC108227409	kinesin-like protein KIN-7K, chloroplastic	K10w
chr7	6410000	exon	DCAR_023880	unknown	K10p
chr7	13030000	intron	LOC108195805	callose synthase 9	K10w
chr7	31840000	exon/3'UTR	LOC108193673	DNA topoisomerase 1-like	K10p
chr8	22950000	3'UTR	LOC108197097	putative pentatricopeptide repeat-containing protein At1g69350, mitochondrial	K10w
chr8	27170000	intron	LOC108198692	uncharacterized	K10w
chr9	5950000	3'URE/exon	LOC108202900	acyl-CoA-binding domain-containing protein 4	K10p
chr9	12810000	3'UTR/intron/exon	LOC108202614	acyltransferase-like protein At1g54570, chloroplastic	K10p
chr9	18090000	intron	LOC108202807	GTPase Der	K10w

Table S15. Insertion sites of *Ivan1* in K10p and K10w callus sub-lines. DH1 reference insertion sites are bolded

Chr.	Beginning of bin	Localization	Gene ID	Gene function	Callus sub-line
**chr1**	**15010000**	**Ref_truncated:intergenic**	**-**	**-**	**K10w,K10p**
chr1	34580000	exon	LOC108204608	rho GTPase-activating protein 5-like	K10w
chr1	34600000	exon	LOC108204459	rho GTPase-activating protein 5-like/filled gap	K10w
chr2	690000	intron/exon	LOC108208035	uncharacterized	K10w
chr2	2490000	intergenic	-	-	K10w
chr2	19750000	intergenicTE/filled gap	-	-	K10w
chr2	19820000	intergenicTE/filled gap	-	-	K10w
chr3	3090000	exon/intergenic/TE	LOC108213968	putative disease resistance RPP13-like protein 3	K10w
chr3	3100000	intergenic/TE	-	-	K10w, K10p
chr3	46040000	exon	LOC108211307	protein trichome birefringence-like 6	K10w
chr4	6580000	intergenic/TE	-	-	K10w, K10p
chr4	6640000	intergenic/TE	-	-	K10w, K10p
chr4	6660000	intergenic/TE	-	-	K10w, K10p
chr4	6680000	intergenic	-	-	K10w, K10p
chr4	12780000	exon	LOC108217144	wall-associated receptor kinase 2-like	K10w
chr4	27060000	intergenicTE/filled gap	-	-	K10w
**chr5**	**35270000**	**Ref_truncated:intergenic/filled gap**	**-**	**-**	**K10w ,K10p**
chr6	18140000	intergenic	-	-	K10w,K10p
chr6	30240000	exon	LOC108226962	vacuolar amino acid transporter 1-like	K10w
chr7	1580000	exon	LOC108193956	probable metal-nicotianamine transporter YSL7	K10w
chr7	1600000	exon	LOC108195326	probable metal-nicotianamine transporter YSL7	K10w
chr7	2180000	intergenic/dcar_023516		hypothetical protein	K10w
chr7	2510000	intergenic	-	-	K10w
**chr8**	**2780000**	**ref:intergenic/TE**	**-**	**-**	**K10w, K10p**

chr8	18250000	intergenic/TE	-	-	K10w
chr8	18280000	intergenic/TE	-	-	K10w
chr8	18330000	intergenic/TE	-	-	K10w
chr8	18350000	intergenic/TE/filled gap	-	-	K10w
chr9	6120000	intergenic	-	-	K10w, K10p

Table S16. Insertion sites of *DcTork_f0/s1917* in K10p and K10w callus sub-lines. DH1 reference insertion sites are bolded

Chr.	Beginning of bin	Localization	Gene ID	Gene function	Callus sub-line
1	26380000	intron/TE	LOC108209641	uncharacterized	K10p
1	46220000	intron	LOC108205248	phosphoribulokinase, chloroplastic-like	K10p
2	21790000	intron/filled gap	LOC108207494	high affinity nitrate transporter 2.4-like	K10p
2	21810000	intergenic/filled gap	-	-	K10p
3	1020000	intergenic/filled gap/TE			K10p
3	8310000	intron/filled gap/TE	LOC108214325	alcohol dehydrogenase-like 7	K10w,K10p
3	34380000	exon	LOC108210590	COP9 signalosome complex subunit 3	K10p
5	8140000	intergenic/TE	-	-	K10p
**5**	**27900000**	**ref:intergenic/filled gap/TE**	**-**	**-**	**K10w,K10p**

Table S17. Insertion sites of *DcTork_f1/s2099* in K10p and K10w callus sub-lines. DH1 reference insertion sites are bolded

Chr.	Beginning of bin	Localization	Gene ID	Gene function	Callus sub-line
**1**	**29450000**	**ref_solo LTR:intergenic/TE**	**-**	**-**	**K10w,K10p**
3	7070000	exon/intron/filled gap	LOC108214349	uncharacterized	K10p
7	2630000	exon	LOC108194228	uncharacterized	K10w,K10p
7	4670000	intergenic/filled gap	-	-	K10w,K10p
**7**	**12410000**	**ref:intergenic**	**-**	**-**	**K10w,K10p**
9	23610000	intergenic/TE	-	-	K10p

Table S18. List of D. *carota* accessions with resequenced genomes. Libraries with insert sizes ranging from 250 to 350 nt were sequenced in the PE mode

No	ID	Taxonomy	Type	Origin	NCBI BioSample no	Specimen Voucher	Coverage	Reference
1	Koral	*D. carota* subsp. *sativus*	eastern cultivated	Poland	SAMN18221085	K10w	~29x	-
2	Koral	*D. carota* subsp. *sativus*	eastern cultivated	Poland	SAMN18221086	K10p	~19x	-
	DH1	*D. carota* subsp. *sativus*	double haploid	Rijk Zwaan	SAMN18221087	DH1do	~28x	-
3	DH1	*D. carota* subsp. *sativus*	double haploid	Rijk Zwaan	SAMN03216637	DH1	~13x	Iorizzo et al. 2016
4	I1	*D. carota* subsp. *sativus*	inbred	USDA	SAMN03766317	B2566B 921-1 USDA	~13x	Iorizzo et al. 2016
5	I2	*D. carota* subsp. *sativus*	inbred	USDA	SAMN03766318	B6274B 927-1 USDA	~13x	Iorizzo et al. 2016
6	I3	*D. carota* subsp. *sativus*	inbred	USDA	SAMN03766319	B7262B 349-1 USDA	~15x	Iorizzo et al. 2016
7	C1	*D. carota* subsp. *sativus*	eastern cultivated	Afghanistan, Badakhshan	SAMN03766321	PI 211590	~12x	Iorizzo et al. 2016
8	C2	*D. carota* subsp. *sativus*	eastern cultivated	China, Beijing	SAMN03766322	PI 652188	~13x	Iorizzo et al. 2016
9	C3	*D. carota* subsp. *sativus*	eastern cultivated	Uzbekistan, Tashkent	SAMN03766323	PI 540422	~12x	Iorizzo et al. 2016
10	C4	*D. carota* subsp. *sativus*	eastern cultivated	Afghanistan	SAMN03766324	PI 200876	~11	Iorizzo et al. 2016
11	C5	*D. carota* subsp. *sativus*	eastern cultivated	Syria, Damascus	SAMN03766325	PI 652336	~13x	Iorizzo et al. 2016
12	C6	*D. carota* subsp. *sativus*	eastern cultivated	Turkey, Mugla	SAMN03766326	PI 652374	~12x	Iorizzo et al. 2016
13	C7	*D. carota* subsp. *sativus*	western cultivated	Japan, Ibaraki	SAMN03766327	PI 652136	~11x	Iorizzo et al. 2016

14	C8	*D. carota* subsp. *sativus*	western cultivated	Brazil, Rio Grande do Sul, Rio Grande	SAMN03766328	Brasilia Embrapa LOTE 39/06	~17x	Iorizzo et al. 2016
15	C9	*D. carota* subsp. *sativus*	western cultivated	Netherlands	SAMN03766329	PI 261648	~15x	Iorizzo et al. 2016
16	C10	*D. carota* subsp. *sativus*	western cultivated	USA, California	SAMN03766330	PI 643114	~16x	Iorizzo et al. 2016
17	C11	*D. carota* subsp. *sativus*	western cultivated	Netherlands	SAMN03766331	PI 451755	~11x	Iorizzo et al. 2016
18	C12	*D. carota* subsp. *sativus*	western cultivated	France	SAMN03766332	PI 264232	~12x	Iorizzo et al. 2016
19	C13	*D. carota* subsp. *sativus*	western cultivated	USA, California	SAMN03766333	PI 632391	~16x	Iorizzo et al. 2016
20	C14	*D. carota* subsp. *sativus*	western cultivated	Belgium	SAMN03766334	PI 187235	~17x	Iorizzo et al. 2016
21	W1	*D. carota* subsp. *carota*	European wild	Portugal, Coimbra	SAMN03766342	PI 502244	~35x	Iorizzo et al. 2016
22	W2	*D. carota* subsp. *carota*	European wild	Portugal, Beja	SAMN03766350	Ames 26408	~28x	Iorizzo et al. 2016
23	W3	*D. carota* subsp. *carota*	European wild	France, Paris	SAMN03766336	PI 478861	~11	Iorizzo et al. 2016
24	W4	*D. carota* subsp. *carota*	Asian wild	China, Xinjiang	SAMN03766338	PI 478369	~17x	Iorizzo et al. 2016
25	W5	*D. carota* subsp. *carota*	Asian wild	Uzbekistan, Gazelkent	SAMN03766335	Ames 27395	~15	Iorizzo et al. 2016
26	W6	*D. carota* subsp. *carota*	Asian wild	Turkey, Konya	SAMN03766343	PI 652393	~38x	Iorizzo et al. 2016
27	W7	*D. carota* subsp. *carota*	Asian wild	Turkey, Izmir	SAMN03766337	PI 652358	~16x	Iorizzo et al. 2016
28	W8	*D. carota* subsp. *carota*	Asian wild	Pakistan, Nomal	SAMN03766339	PI 274297	~46x	Iorizzo et al. 2016

29	Ssp1	*D. carota* subsp. *gummifer*	European wild	Portugal, Faro	SAMN03766344	Ames 26381	~32x	Iorizzo et al. 2016
30	Ssp2	*D. carota* subsp. *gummifer*	European wild	Portugal, Faro	SAMN03766345	Ames 26383	~22x	Iorizzo et al. 2016
31	Ssp3	*D. carota* subsp. *gummifer*	European wild	France	SAMN03766351	Ames 31194	~24x	Iorizzo et al. 2016
32	Ssp4	*D. carota* subsp. *gummifer*	European wild	France, Finistere, Le France, Conquet	SAMN03766341	PI 478883	~43x	Iorizzo et al. 2016
33	Ssp5	*D. carota* subsp. *capillifolius*	European wild	Libya, Jefren	SAMN03766340	PI 279764	~36x	Iorizzo et al. 2016

Table S19. Summary of verification of *de novo* insertion sites of *Alex1* in K10p and K10w callus sub-lines

Chr.	Beginning of bin	PCR verification	Primers used for verification of *de novo* insertions	Expected length of product with insertion [bp]	Sanger-sequenced
chr1	1480000	+	Alex1_chr1_1488877_F + Alex1_R	1501	no
chr1	38430000	+	Alex1_chr1_38432446_F + Alex1_R	767	yes
chr3	9950000	+	Alex1_chr3_9958916_R + Alex1_R	619	no
chr4	4750000	+	Alex1_chr4_4759530_R + Alex1_R	1140	no
chr4	24970000	+	Alex1_chr4_24972935_F + Alex1_R	697	no
chr5	9000000	+	Alex1_chr5_9005708_F + Alex1_F	1189	no
chr6	13140000	+	Alex1_chr6_13148731_R + Alex1_R	617	yes
chr7	13170000	+	Alex1_chr7_13174817_F + Alex1_R	731	no
chr8	11870000	+	Alex1_chr8_11878594_R + Alex1_R	776	yes

Table S20. Summary of verification of *de novo* insertion sites of *Alex3* in K10w and K10p callus sub-lines

Chr.	Beginning of bin	PCR verification	Primers used for verification of *de novo* insertions	Expected length of product with insertion [bp]	Sanger-sequenced
chr1	8740000	+	Alex3_chr1_8745037_F + Alex3_R	668	no
chr2	590000	+	Alex3_chr2_596095_F + Alex3_R	705	no
chr4	15450000	+	Alex3_chr4_15457770_F + Alex3_R	986	no
chr4	24940000	+	Alex3_chr4_24949993_F + Alex3_R	722	yes
chr4	32110000	+	Alex3_chr4_32110803_R + Alex3_R	673	no
chr6	34050000	+	Alex3_chr6_34059013_F + Alex3_R	482	yes
chr6	34960000	+	Alex3_chr6_34963662_R + Alex3_R	886	yes
chr9	18090000	+	Alex3_chr9_18096966_F + Alex3_R	674	no

Table S21. Primers for verification of LTR-RT circularization

Primer name	Primer sequence (5'→3')	Length of eccDNA product (bp)
Alex1_eccDNA_F	CCATCAACAAAGCCCCATAA	1052
Alex1_eccDNA_R	TAATCCTACTCCACTCCATGC	
Alex3_eccDNA_F	AATTTTTGCCGTTTAGGCGA	750
Alex3_eccDNA_R	CCACTACTCTACAGGTTGCT	

Table S22. Primers for verification of the presence of the LTR-RTs domain transcripts and for RT-qPCR analysis

Primer name	Primer sequence (5'→3')	Length of cDNA product (bp)	Primer efficiency (%)	R^2
Alex1_cDNA_RVT2_F	GGTCATTTTAGCAACTGGTG	114	90.7	0.999
Alex1_cDNA_RVT2_R	CTCAAGTCTGATGGCTCATT			
Alex3_cDNA_rve_F	TTCACCCCCATTATCAGACC	109	100.0	1.000
Alex3_cDNA_rve_R	GCTTCGTTCCAAATCTGAAG			

Table S23. Primers for verification of *de novo* insertions of *Alex1*. Primers for the reference insertion site are bolded

Primer name	Primer sequence (5'→3')	Product length (bp)
Alex1_F	CCATCAACAAAGCCCCATAA	specific to *Alex1*
Alex1_R	TAATCCTACTCCACTCCATGC	
Alex1_chr1_1488877_F	ATCACCATCAAAGCATCGTC	998
Alex1_chr1_1488877_R	TGACTAAAGAGCAGACAGGA	
Alex1_chr1_38432446_F	TCCAATACAACTACCTGCCG	354
Alex1_chr1_38432446_R	TCTTAGTCTCAGGTTATCGCC	
Alex1_chr3_9958916_F	GACCATCACTATTTTTGGAACA	211
Alex1_chr3_9958916_R	CTTGCCTTTAAGAGGTGACTT	
**Alex1_chr3_27717891_F**	**AGCGTACCAGCCAAAATTGAG**	**770**
**Alex1_chr3_27717891_R**	**TAATCCTACTCCACTCCATGC**	
Alex1_chr4_4759530_F	ATTTTATGAGATACGGTGCGA	2357
Alex1_chr4_4759530_R	TGTCTTATCCCCAGAACGAT	
Alex1_chr4_24972935_F	TGCCTACACCCAGATCAAT	2070
Alex1_chr4_24972935_R	ATTTTCTTTGACCGACTCGTTAG	
Alex1_chr5_9005708_F	CTTAGATGTCGCAGATTCATCG	1697
Alex1_chr5_9005708_R	AGTGACATAAAGGATGTGAAGC	
Alex1_chr6_13148731_F	GTTCTGTTCGCTGGATACAAG	819
Alex1_chr6_13148731_R	TGTCAAGATCATTGATAGCCT	
Alex1_chr7_13174817_F	TCCAAGGCTTTGCGGTAATA	306
Alex1_chr7_13174817_R	TCTTGAGCGAATGAAGTGTAA	
Alex1_chr8_11878594_F	AAAGATACCGTGAACCGATAC	1036
Alex1_chr8_11878594_R	CCACAATGACTGTGTTGTTCTA	

Table S24. Primers used for verification of *de novo* insertions of *Alex3*. Primers for the reference insertion site are bolded

Primer name	Primer sequence (5'→3')	Product length (bp)
Alex3_R	CCACTACTCTACAGGTTGCT	specific to *Alex3*
Alex3_chr1_8745037_F	GTGGGATGACTCCTAGAGTTT	527
Alex3_chr1_8745037_R	TCTTTTCCTCTCTGCGTGC	
Alex3_chr2_596095_F	TCTTGGTTCTCTTGTTTTATTGC	877
Alex3_chr2_596095_R	CAGCCCTTCTCTGGATAACTTC	
**Alex3_chr3_3148385_F**	**AGGCGACTATATACAGCTTAGGG**	**1536**
**Alex3_chr3_3148385_R**	**CCACTACTCTACAGGTTGCT**	
Alex3_chr4_15457770_F	CTATTCTCCTCGGGAACTACAG	1295
Alex3_chr4_15457770_R	CCAACCAAGATTAGTGGGC	
Alex3_chr4_24949993_F	AATGATTTGGGGACACTTACAT	806
Alex3_chr4_24949993_R	TTATTGTTCAGCAGAACTGGG	
Alex3_chr4_32110803_F	CGATCAAGGAACTATCCACG	527
Alex3_chr4_32110803_R	CAGCCACAAGCCAATGATAG	
Alex3_chr6_34059013_F	CATACTAAAACCATCCTCTGGC	217
Alex3_chr6_34059013_R	TGGGAACTTCTCTGATTGATG	
Alex3_chr6_34963662_F	CGAGAAAAATGCTTCTGATAGG	641
Alex3_chr6_34963662_R	AGCGACGTAAGAAATAAGGTT	
Alex3_chr9_18096966_F	TTCTTACGAAGCCAGTCCC	1697
Alex3_chr9_18096966_R	TAGTTGACGAACCTGGAGTTA	
